# SoyCountNet: a deep learning framework for counting and locating soybean seeds in field environment

**DOI:** 10.3389/fpls.2026.1743104

**Published:** 2026-02-25

**Authors:** Fei Liu, Qiong Wu, Haoyu Wang, Zhongzhi Han, Shudong Wang, Longgang Zhao, Zhaohua Wang, Hexiang Luan

**Affiliations:** 1 College of Science and Information Science, Qingdao Agricultural University, Qingdao, China; 2 Qingdao Institute of Software, College of Computer Science and Technology, China University of Petroleum (East China), Qingdao, China; 3 Institute of Agricultural Information and Economics, Shandong Academy of Agricultural Sciences, Jinan, China

**Keywords:** attention mechanism, deep learning, point-to-point network, precision breeding, seeds per plant

## Abstract

**Introduction:**

Accurate counting and spatial localization of soybean seeds—particularly Seeds Per Plant (SPP)—are critical for yield estimation and cultivar evaluation. In field environments, however, complex backgrounds, pod occlusion, and uneven grain filling make high-precision counting challenging, and traditional methods often struggle to balance accuracy and robustness.

**Methods:**

To address these challenges, this study proposes SoyCountNet, a deep learning framework for automatic soybean seed counting and localization at the single-plant level under field conditions. The model is built on a self-constructed field-based phenotyping platform and optimized using the lightweight Point-to-Point Network (P2PNet). For feature extraction, a VGG19_BN backbone and a Super Token Sampling Vision Transformer (SViT) module are employed to enhance local feature representation and global contextual understanding. During feature fusion, the Efficient Channel Attention (ECA) mechanism strengthens seed-related features while suppressing interference from leaves, stems, and soil. Furthermore, an improved loss function that combines point-distance constraints with overlap penalties enhances both counting precision and spatial consistency.

**Results:**

Experimental results demonstrate that SoyCountNet outperforms existing approaches on the field soybean dataset. It achieves a mean absolute error (MAE) of 4.61, a root mean square error (RMSE) of 6.03, and a coefficient of determination (R²) of 0.94. The model demonstrates consistent performance across the tested soybean cultivars, providing reliable SPP estimates within the evaluated dataset.

**Discussion:**

These findings indicate that SoyCountNet offers a reliable and scalable solution for precise soybean seed counting and localization in complex field environments. Its lightweight architecture allows deployment on intelligent agricultural platforms, supporting high-throughput phenotyping, yield prediction, and precision breeding, while providing a foundation for the future development of intelligent and sustainable agricultural technologies.

## 1 Introduction

With the rapid development of smart agriculture and high-throughput phenotyping (HTP) technologies, automated and precise acquisition of crop phenotypic data has become essential for modern breeding and yield prediction (Fan et al., 2021; Zavafer et al., 2023; Liu et al., 2025b). Soybean (Glycine max), valued for its high protein and oil content, is an economically and ecologically important crop (Mishra et al., 2024). Soybean yield is influenced by multiple agronomic traits, including pod number, seed number, and their spatial distribution, which serve as key indicators for evaluating population productivity and guiding genetic improvement (Zhao et al., 2023; Yang et al., 2024; Zhang et al., 2025). Among these, the number of seeds per plant is a particularly critical determinant of yield (Li et al., 2020; Yang et al., 2024). Traditional seed counting methods, relying on manual observation or semi-automated image analysis, are time-consuming, labor-intensive, and prone to human error. Under complex field conditions—characterized by variable lighting, severe occlusion, heterogeneous backgrounds, and incomplete grain filling—manual counting often results in substantial inaccuracies (Wattana et al., 2018). Recent advances in computer vision and deep learning (DL) have provided effective tools to overcome these challenges.

In recent years, DL has demonstrated substantial potential in agricultural computer vision, offering robust solutions for crop phenotyping, object counting, and localization under complex field conditions (Murphy et al., 2024; Jin et al., 2025). For instance, region-based detectors such as Faster R-CNN achieve high precision in localizing fruits and plant organs (Behera et al., 2023; Tian et al., 2025), whereas single-stage models like YOLO and SSD provide a favorable balance between accuracy and computational efficiency in high-throughput counting tasks (Li et al., 2024; Yu et al., 2024). However, anchor-based detection frameworks often encounter difficulties when dealing with dense, small, or occluded targets, resulting in missed detections, redundant predictions, and localization errors. These limitations constrain their applicability in high-density crop counting and fine-grained phenotyping. To address these challenges, recent studies have explored point-based counting strategies and attention-enhanced architectures to improve the detection of small, dense, and occluded targets (Wu et al., 2025; Jin et al., 2025).

DL has also been increasingly applied to soybean phenotyping, improving automation and accuracy in feature extraction from complex field images (Okada et al., 2024; Kwon et al., 2025). These advancements have facilitated high-throughput data acquisition and data-driven decision-making in breeding, germplasm improvement, and yield prediction (Zhou et al., 2025). For example, Moeinizade et al. (2022) employed a CNN–LSTM model with UAV-based time-series imagery to estimate soybean maturity, while Li et al. (2024) proposed SoybeanNet, which integrates a Transformer with point regression for pod counting and localization. Chen et al. (2023) utilized hyperspectral imaging to quantify leaf traits, and Wei et al. (2023) applied Kinect 2.0–based 3D reconstruction to estimate the leaf area index (LAI). Zhang Z, et al. (2024) developed DSBEAN to link phenotypes with genetic improvement, and (Liu et al., 2025a) introduced SmartPod for automatic pod counting under field conditions. Despite these advances, automatic seed counting at the single-plant level remains largely underexplored. Most existing statistical or vision-based approaches exhibit limited robustness in complex field environments, where heterogeneous backgrounds, dense occlusion, and subtle seed textures degrade detection accuracy and spatial consistency. Therefore, developing a robust and scalable DL framework for accurate seed counting under real-world field conditions is essential for intelligent soybean phenotyping and precision breeding.

To address this gap, this study constructed a point-annotated soybean seed dataset using a self-developed field phenotyping platform and proposed SoyCountNet, a deep learning framework built upon the lightweight P2PNet. The framework adopts a multi-level feature optimization strategy: the VGG19_BN backbone enhances feature representation and training stability, while the SViT module captures both local and global contextual information to improve the detection of dense and occluded seeds. During feature fusion, the ECA mechanism highlights seed-related features and suppresses background interference. Moreover, an improved loss function combining Nearest-Neighbor and Target Overlap penalties mitigates uneven point distribution and redundant predictions, thereby enhancing localization consistency and counting robustness. Experimental results demonstrate that SoyCountNet significantly improves seed detection and counting accuracy under complex field conditions while maintaining a lightweight and deployable architecture, providing a scalable solution for intelligent field phenotyping and high-throughput seed counting.

## 2 Materials and methods

### 2.1 Experimental conditions, data acquisition, and annotation

**Figure 1:**
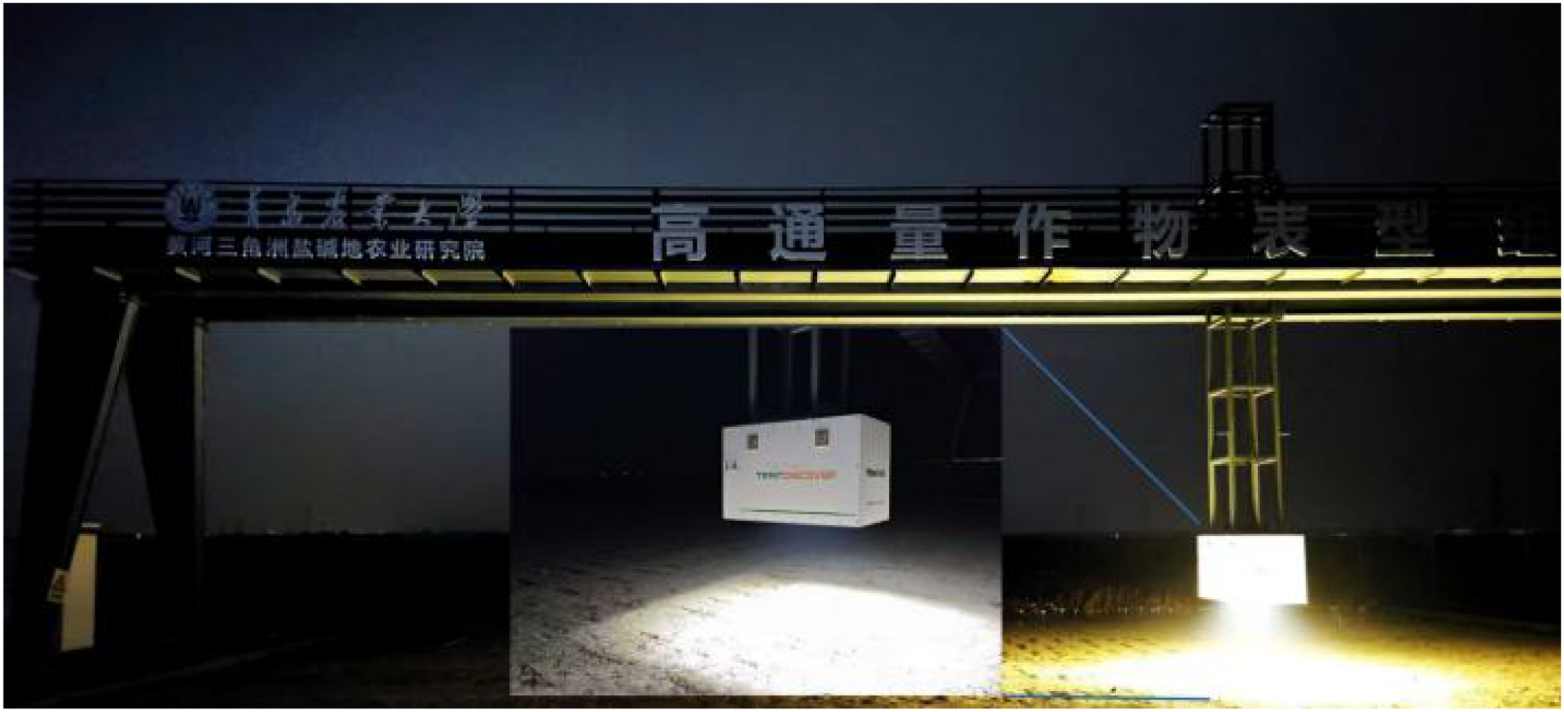
Schematic diagram of the TraitDiscover high-throughput phenotyping platform.

Data were acquired using the automated gantry-based field phenotyping system TraitDiscover, equipped with an industrial-grade Trait-RGB camera, as shown in **Figure 1**, with main specifications listed in **Table 1**. The system supports high-throughput, multi-angle imaging with controlled illumination and automatic positioning, enabling continuous and precise capture of crop samples. The camera was mounted at a fixed height of 1.8 m to capture the full canopy of each soybean plant. To minimize the influence of natural light fluctuations, all images were collected at night under uniform illumination provided by the platform’s built-in constant light source. Each plant was photographed from both front and back views to enhance target coverage and counting accuracy.

**Table 1:** Parameters of the Trait-RGB.

Field of view	2000mm*1500mm@H=1500mm	Camera sensor type	CMOS
Pixel Resolution	6460*4850(3100 Megapixels)	Frame Rate	8fps
Focal Length	8mm	Color Type	RGB Color
Lens Mount	C-Mount	Pixel Size	3.45um*3.45um
Object Distance	2000mm~3500mm	Exposure Time	46us-2sec
Camera Sensor Size	≥14.1mm*10.3mm	Imaging Light Source	Four-band LED supplemental lighting, standard color calibration with a color reference card

**Figure 2:**
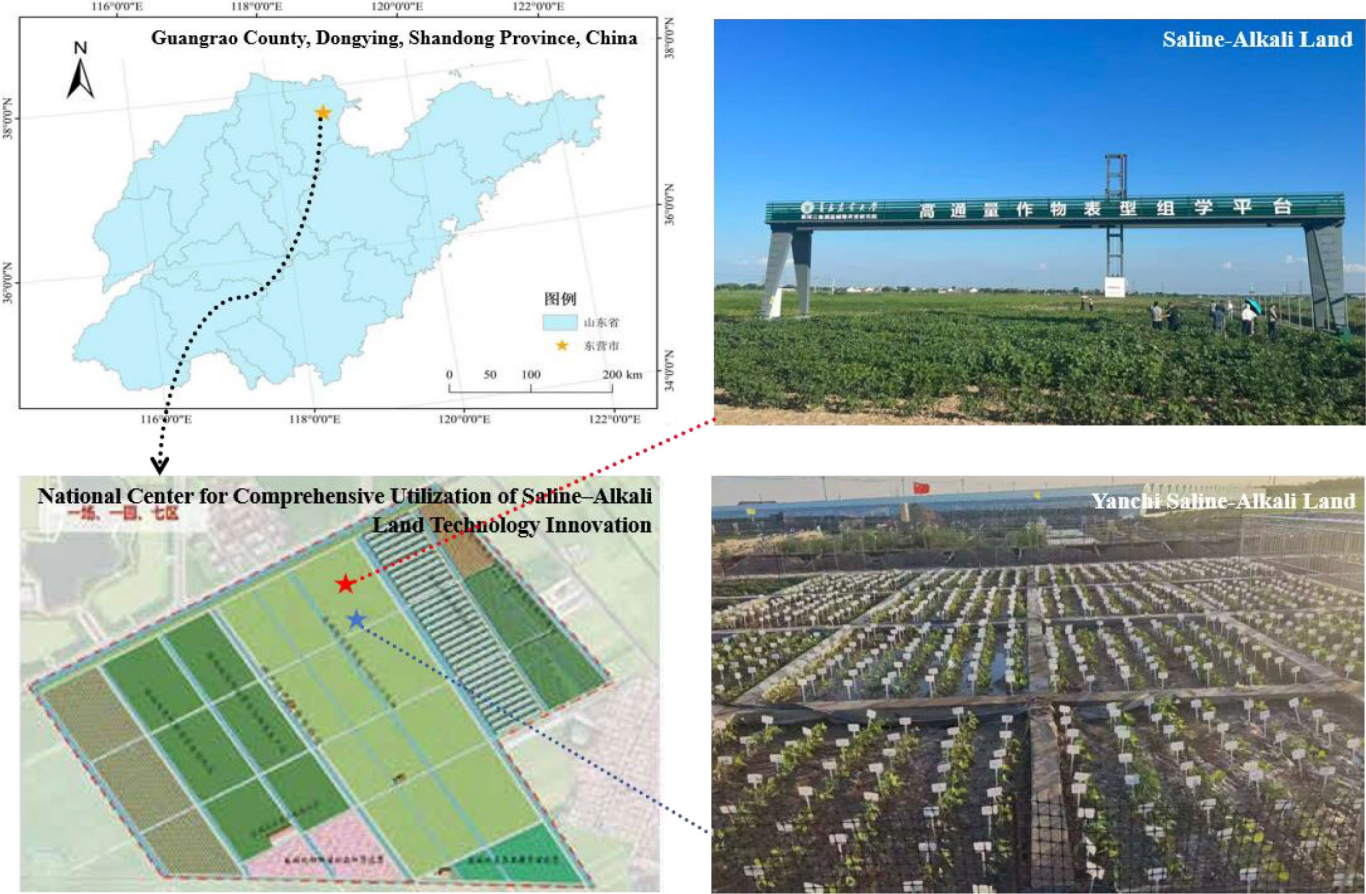
Geographical location of the experimental fields.

The field experiment was conducted from June 2024 to October 2025 at the National Saline–Alkali Land Comprehensive Utilization Technology Innovation Center in Guangrao County, Dongying, Shandong Province, China (37°18′36″ N, 118°39′0″ E), as shown in **Figure 2**. The field layout followed a standardized planting scheme with 40 cm row spacing and 10 cm plant spacing. To simulate different salinity conditions, the soil was treated with 0‰ NaCl for the control group and 2.5‰ NaCl for the stress group. Six representative soybean cultivars were cultivated independently under both conditions, with an equal number of images collected under each treatment (1:1 ratio). A total of 800 high-resolution RGB images were obtained at the mature stage, covering diverse cultivars and plant architectures. All images were divided into training, validation, and test sets in an 8:1:1 ratio, with 20 images from each cultivar selected for cross-cultivar robustness evaluation.

**Figure 3:**
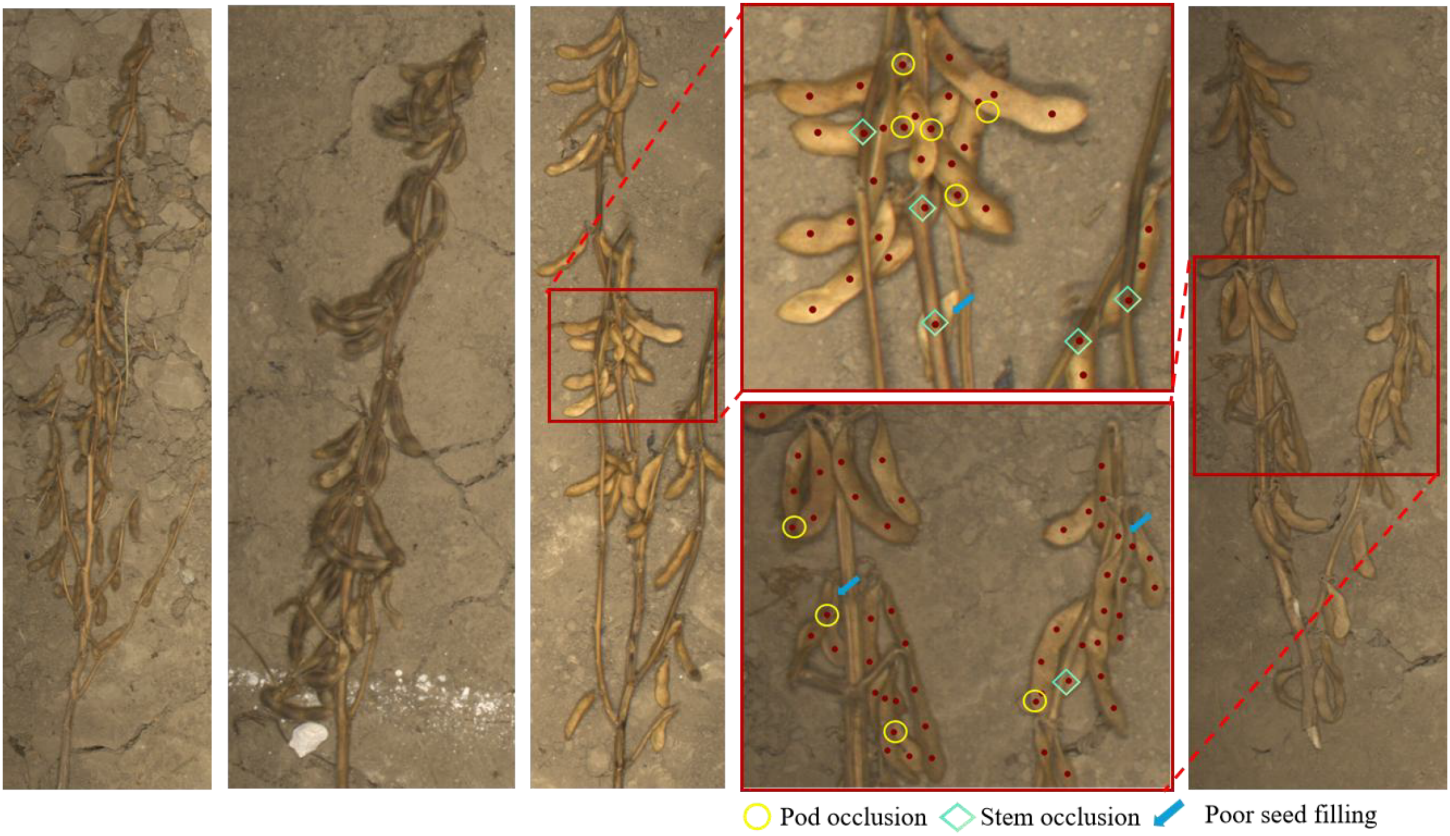
Representative single-plant soybean images and corresponding point annotations across different cultivars. The examples illustrate variations in background complexity, including pod and stem occlusion, similar soil coloration, and incomplete pod filling.

For seed-level annotation, a point-based labeling strategy was adopted, where each soybean seed was marked by a single pixel at its centroid. Three researchers with agronomy expertise independently performed the annotations, which were cross-verified by an expert to minimize subjective bias. For overlapping or partially occluded seeds, only the visible central region was annotated to avoid redundant markings. To ensure annotation consistency, a quality control protocol combining cross-validation and random inspection was applied: after every 100 annotated images, 10% were randomly reviewed, and any coordinates deviating by more than 8 pixels or showing missing or duplicate labels were corrected. The final mean annotation error was controlled within 6 pixels. Annotation data were stored in TXT format, including image paths, seed coordinates, cultivar, and salinity condition for subsequent model training and supervision. Representative examples of the annotated data are presented in **Figure 3**.

**Figure 4:**
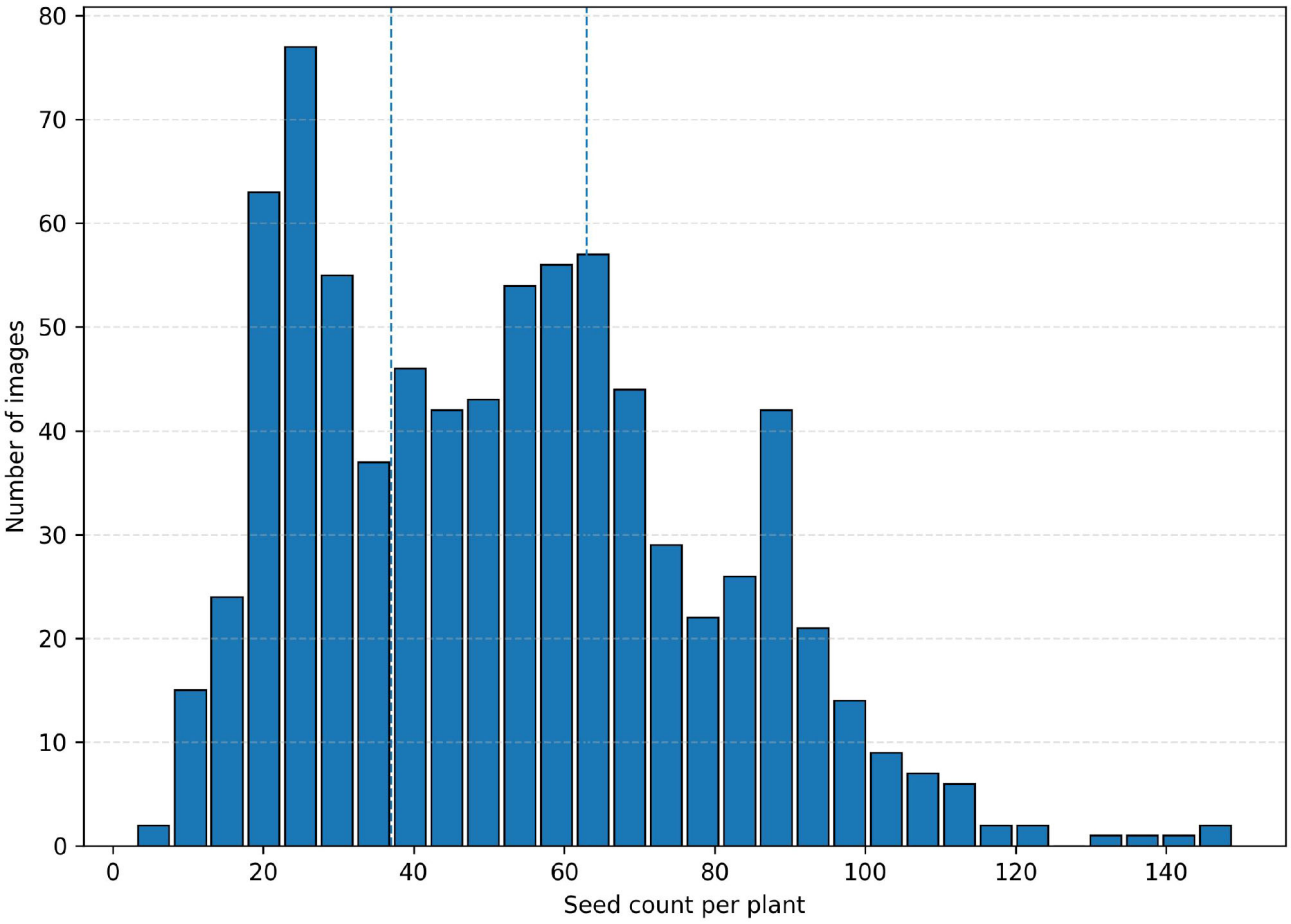
Distribution of seed counts per plant in the constructed dataset. The dashed vertical lines indicate the quantile-based thresholds used for coarse density stratification.

The number of seeds per plant was counted for all 800 images, with a minimum of 3 seeds, a maximum of 149 seeds, and an average of 52.33 seeds per plant (see **Table 2**), indicating substantial variation in seed density among the samples. From the perspective of the counting task, **Figure 4** presents the distribution histogram of seed counts per plant. The results show that the seed counts exhibit a continuous range from sparse to dense, rather than being concentrated within a narrow interval. Combined with the density stratification statistics in **Table 2**, the dataset includes 273 sparse samples (≤37 seeds), 267 medium-density samples (38–63 seeds), and 260 dense samples (>63 seeds), providing a relatively balanced distribution across different density levels.

**Table 2:** Statistical summary of seed counts and density stratification in the dataset.

Number of images	Min seeds/plant	Mean seeds/plant	Max seeds/plant	Sparse (≤ 37 seeds)	Medium (38–63 seeds)	Dense (> 63 seeds)
800	3	52.22	149	273	267	260

### 2.2 SoyCountNet for soybean seed counting and localization

An improved end-to-end counting and localization framework, SoyCountNet, was developed based on the P2PNet architecture to achieve high-precision seed counting and stable localization under field conditions. The framework addresses challenges arising from complex backgrounds, pod occlusion, and uneven seed filling. Unlike conventional density- or bounding-box-based methods, P2PNet directly learns the spatial distribution of target centers from point annotations by regressing their coordinates, avoiding blurred density estimation and feature drift in high-density or occluded scenarios. Its architecture comprises a backbone network, a feature fusion layer, and a point regression branch, optimized with a multi-task loss function to minimize spatial deviations between predicted and ground-truth points. This design enables end-to-end training, maintains a lightweight structure, and ensures high spatial localization accuracy (Zhao et al., 2023).

Soybean seed images collected under field conditions often exhibit complex textures, similar foreground–background colors, cultivar-specific morphological variations, and weak seed bulging features, which can limit the accuracy and robustness of the original P2PNet. Duplicate, missing, or mislocalized predictions are particularly common in high-density, overlapping, or small-object scenarios. To overcome these limitations, SoyCountNet incorporates four key enhancements:

1. Backbone Feature Extraction (VGG19_BN): efficient multi-scale spatial encoding for enhanced feature representation.
2. Global Context Modeling (SViT): captures long-range dependencies and global semantic information.
3. Feature Enhancement (ECA): amplifies discriminative seed features while suppressing background interference.
4. Loss Function: combines point-distance constraints and overlap penalties to improve counting precision and spatial consistency.

These modules preserve the lightweight, end-to-end design of P2PNet while substantially improving counting accuracy and robustness under complex field conditions.

**Figure 5:**
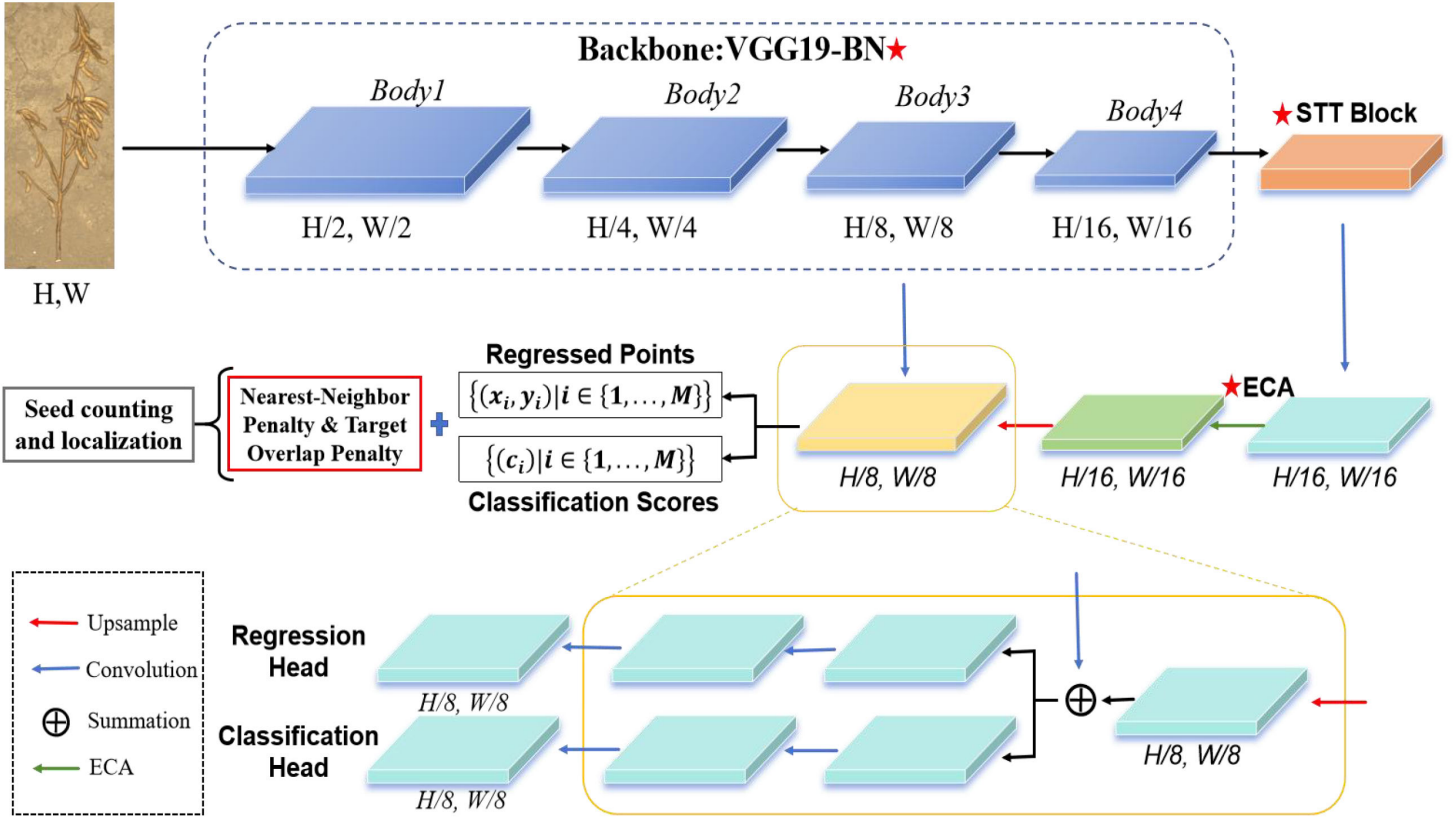
The SoyCountNet framework for soybean grain counting and localization.

The processing workflow of SoyCountNet is as follows. Annotated RGB images are first passed through the VGG19_BN backbone to extract high-level features with rich local semantics. The SViT module then models global context, capturing long-range dependencies and semantic relationships, which is particularly beneficial in dense or occluded regions. Extracted features are subsequently enhanced via ECA, which emphasizes seed-specific channel responses while suppressing interference from leaves, stems, and soil, thereby improving localization accuracy and counting stability. Finally, regression and classification heads predict spatial coordinates and confidence scores for each seed. During training, the loss function incorporates Nearest-Neighbor and Target Overlap penalties to regulate spatial distribution, reduce duplicate predictions, and minimize mismatches. Through this hierarchical and modular design, SoyCountNet achieves fast, accurate, and robust soybean seed counting and localization under complex field conditions, providing a reliable solution for high-throughput phenotyping and yield estimation. The overall architecture is illustrated in **Figure 5**.

#### 2.2.1 VGG19_BN

In the SoyCountNet framework, VGG19_BN serves as the backbone network to extract multi-scale convolutional features from input images, providing a foundation for subsequent tasks. Based on the classical VGG19 architecture, it consists of 16 convolutional layers organized into five convolutional blocks. Each block contains 2–4 consecutive 3×3 convolutional layers followed by ReLU activation, with batch normalization (BN) added after each block to enhance training stability, accelerate convergence, and improve feature consistency. Layer-wise convolution and pooling progressively expand the receptive field, enabling features to capture information at multiple scales. Low-level features encode textures, edges, and local morphology; mid-level features capture both local patterns and partial context; and high-level features provide semantic understanding in dense or complex regions. These multi-scale feature maps are essential for high-precision point detection: low-level features support edge detection of small seeds, mid-level features balance local texture and contextual information, and high-level features supply semantic cues to distinguish dense or occluded targets. The deep convolutional structure enhances feature representation, enabling stable performance in field images with complex backgrounds, similar soil colors, large pod shape variations, or subtle seed features. In SoyCountNet, outputs from VGG19_BN are fed into both the SViT module for global context modeling and the ECA module for channel-wise attention. Through multi-level feature fusion and channel-weighted enhancement, the framework significantly improves single-seed localization accuracy and robustness, providing a solid foundation for dense, multi-scale soybean seed detection under complex field conditions.

#### 2.2.2 Super vision transformer

**Figure 6:**
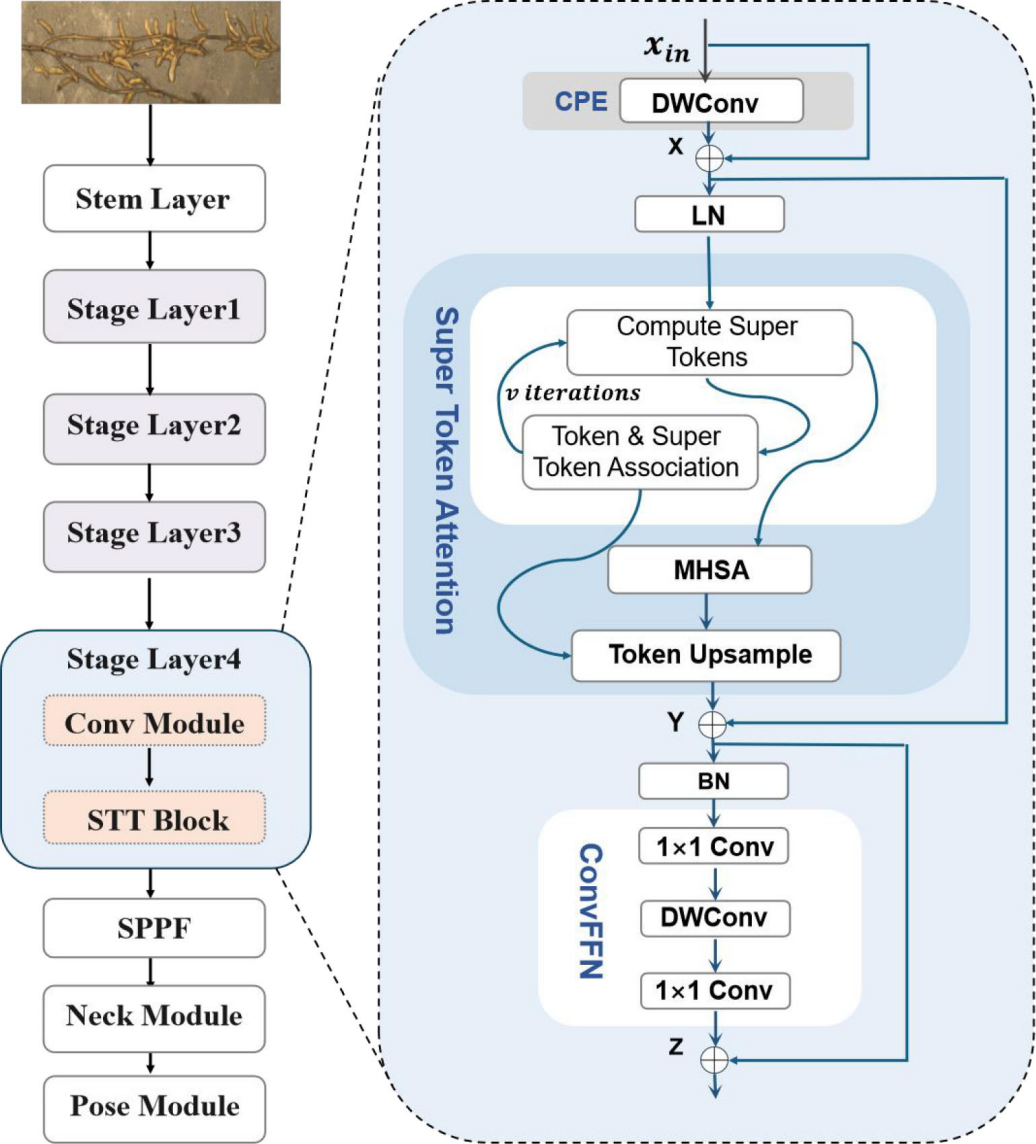
Structure of SViT Block.

In point detection tasks, conventional convolutional neural networks (CNNs) are effective at capturing local features but are limited by restricted receptive fields, which hinder the modeling of long-range dependencies and global context. This limitation is particularly pronounced in field images of densely packed or occluded soybean seeds. To address this issue, the SViT module (Han et al., 2022) was incorporated after the VGG19_BN backbone in SoyCountNet. SViT tokenizes high-level convolutional features and aggregates semantically related tokens, enabling joint modeling of local details and global context. This design improves the detection of dense, small, and occluded seeds. As illustrated in **Figure 6**, SViT consists of three core components:

1. Convolutional Position Embedding (CPE): encodes spatial information before tokenization to preserve positional structure, allowing the Transformer to distinguish local features at different locations.
2. Super Token Attention (STA): aggregates semantically similar tokens into “super tokens,” integrating local features and modeling global dependencies in dense target regions while suppressing background interference.
3. Convolutional Feed-Forward Network (ConvFFN): enhances feature representation after attention, preserving spatial structure and combining global semantics with local details for subsequent point regression and classification.

The globally enhanced features output by SViT are then fed into the ECA module for channel weighting, emphasizing discriminative seed features while suppressing interference from leaves, stems, and soil. This provides high-quality input for the regression and classification branches. Experimental results demonstrate that SViT significantly improves the spatial distribution accuracy of predicted points and counting robustness under high-density, occlusion, and complex background conditions. Furthermore, the module maintains the lightweight design of the model, enhancing SoyCountNet’s adaptability and generalization for multi-scale soybean seed detection in complex field environments.

#### 2.2.3 Efficient channel attention

Soybean seed images often contain stems, leaves, pods, and soil-colored regions, which introduce irrelevant features and reduce the model’s discriminative capability. To emphasize salient seed-related channels while suppressing background noise, SoyCountNet incorporates the ECA module after feature extraction and convolution operations (Wang et al., 2020b). The core mechanism of ECA is the adaptive modeling of inter-channel dependencies via a one-dimensional convolution. Unlike conventional channel attention mechanisms that rely on fully connected layers for dimensionality reduction and expansion, ECA performs local convolution directly along the channel dimension, preserving inter-channel relationships while substantially reducing computational complexity. Specifically, ECA adaptively determines the kernel size k based on the number of input channels and computes the relative importance of each channel, dynamically adjusting their contribution to the overall feature representation, the formula is shown in Equation 1.

(1) $$k=\psi (C)={\left|\frac{\log_{2}(C)}{\gamma }+\frac{b}{\gamma }\right|}_{odd}$$

Where *C* denotes the number of input feature channels and ${\left|t\right|}_{odd}$ is the odd integer closest to *t*.

**Figure 7:**
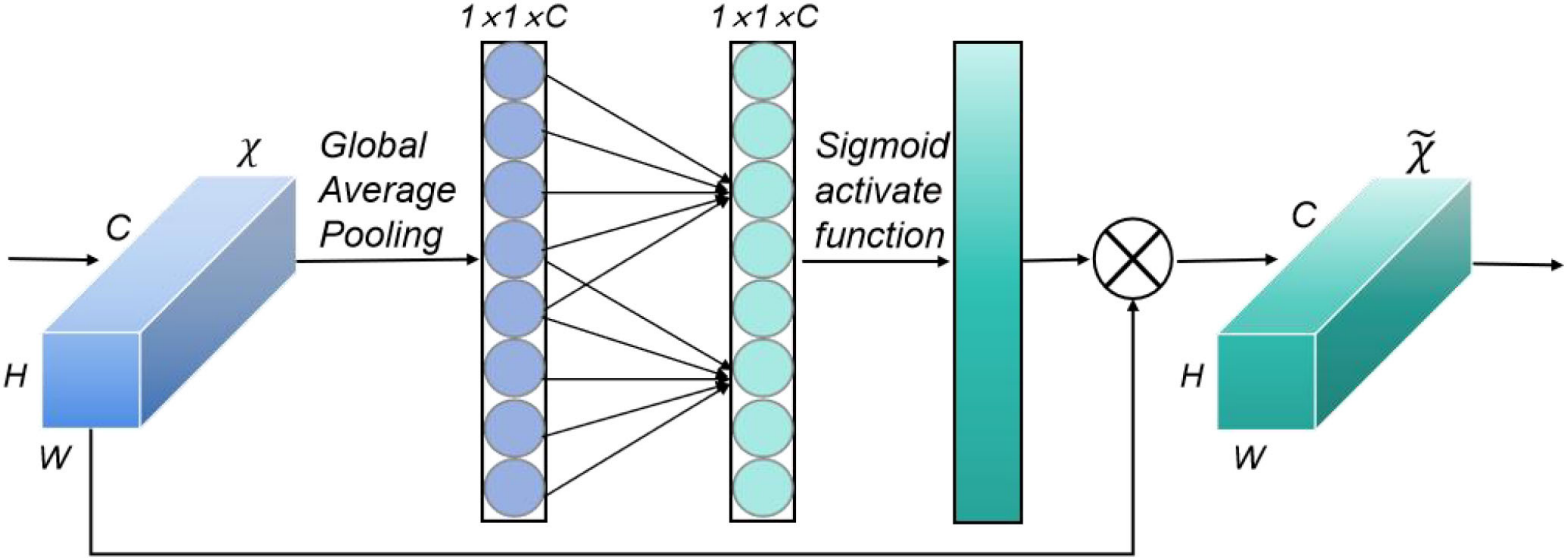
Architecture of the ECA module.

This mechanism strengthens the response of salient seed channels, enhancing both the localization accuracy and counting robustness of the point-detection branch, particularly under dense, occluded, or morphologically similar targets. When integrated with multi-level features from the VGG19_BN backbone and SViT module, ECA effectively optimizes global feature representation, achieving efficient channel attention without significantly increasing model parameters. Experimental results demonstrate that ECA substantially improves the spatial distribution accuracy of predicted points and enhances counting robustness under complex field conditions. The structural overview of the module is illustrated in **Figure 7**.

#### 2.2.4 Loss function

The loss function is a core component of SoyCountNet, directly influencing both localization accuracy and counting performance. In the original P2PNet, classification loss and point regression loss are jointly optimized to achieve target detection and localization. However, for field images of densely packed or heavily occluded soybean seeds, the original loss may lead to duplicate predictions or positional deviations, limiting counting accuracy and robustness. To address these issues, SoyCountNet introduces two additional penalty terms: the Nearest-Neighbor Penalty and the Overlap Penalty, which constrain the spatial proximity of predicted points and the degree of overlap, respectively. These penalties enhance localization stability and counting accuracy under complex field conditions. The overall loss function is expressed as Equation 2.

(2) $$L_{total}=L_{cls}+\lambda_{reg}L_{reg}+\lambda_{near}L_{near}+\lambda_{overlap}L_{overlap}$$

Where $L_{cls}$ denotes the classification loss, $L_{reg}$ denotes the point regression loss, $L_{near}$ represents the nearest-neighbor penalty, and $L_{overlap}$ represents the overlap penalty. The coefficients $\lambda_{reg}$, $\lambda_{near}$ and $\lambda_{overlap}$ are used to balance the contributions and relative weights of each loss term.

The classification loss and regression loss constitute the basic supervision component, the formula is shown in Equation 3.

(3) $$L_{base}=L_{cls}+\lambda_{reg}L_{reg}$$

Where $L_{cls}$ employs binary cross-entropy loss to distinguish foreground from background, while $L_{reg}$ uses the Smooth L1 loss function to constrain the distance between predicted points and ground-truth points.

To prevent the model from generating excessive and overly close predicted points around the same target, the Nearest-Neighbor Penalty introduces a constraint on the spatial distribution of predicted points. Let the set of predicted points be $p={\left\{p_{i}\right\}}_{i=1}^{N_{p}}$, and the Euclidean distance between any two points is defined as Equation 4.

(4) $$d_{ij}={\left\|p_{i}-p_{j}\right\|}_{2}$$

A penalty is applied when $d_{ij}<d_{\min}$, defined as Equation 5.

(5) $$L_{near}=\frac{1}{N_{p}^{2}}\sum_{i\neq j}\max(0,d_{\min}-d_{ij})$$

This term penalizes predicted points that are too close to each other, effectively reducing duplicate detections, with its effect controlled by the coefficient $\lambda_{\text{near}}$. Meanwhile, the overlap penalty is applied to mitigate excessive clustering of predictions around a single ground-truth point. Let the set of ground-truth points be $G={\left\{g_{i}\right\}}_{i=1}^{N_{g}}$, and let $n_{i}$denote the number of predicted points within a distance $g_{i}$less than the threshold $r_{o}$. The overlap penalty is then defined as Equation 6.

(6) $$L_{overlap}=\frac{1}{N_{g}}\sum_{i=1}^{N_{g}}\max(0,n_{i}-1)$$

This term constrains the redundant distribution of predicted points around a single target, ensuring that each ground-truth point corresponds to only one prediction.

By integrating the Nearest-Neighbor Penalty and Overlap Penalty into the original loss function, SoyCountNet effectively regulates the spatial distribution of predicted points, reducing duplicate detections and overmatching. The weighting coefficients and distance thresholds in the loss function were determined based on training set observations and prior knowledge of point-based counting methods. The weights of the nearest-neighbor and overlap penalties provide sufficient regularization without overshadowing the classification and regression losses. Distance thresholds were set according to the average spatial scale of soybean seeds, triggering penalties only when predicted points are excessively close or clustered. This enhancement significantly improves the model’s robustness and counting accuracy in dense or occluded scenarios, while ensuring stable and convergent training and providing a solid foundation for subsequent network optimization and parameter tuning.

#### 2.2.5 Evaluation metrics

To comprehensively evaluate the proposed soybean seed counting framework, multiple aspects were considered, including counting accuracy, robustness, model fit, and statistical reliability. The specific evaluation metrics are as follows:

1. Mean Absolute Error (MAE): quantifies the average absolute deviation between predicted and manually counted values, the formula is shown in Equation 7.

(7) $$MAE=\frac{1}{N}\sum_{i=1}^{N}\left|y_{i}-{\hat{y}}_{i}\right|$$

2. Root Mean Square Error (RMSE): measures the deviation between predicted and manually counted values, reflecting the robustness of predictions, the formula is shown in Equation 8.

(8) $$MSE=\frac{1}{N}\sum_{i=1}^{N}{\left(y_{i}-{\hat{y}}_{i}\right)}^{2},RMSE=\sqrt{MSE}$$

3. Coefficient of Determination ( $R^{2}$): quantifies the proportion of variance in the observed data explained by the predictions. A $R^{2}$value closer to 1 indicates a better fit between the model and the data, the formula is shown in Equation 9.

(9) $$R^{2}=1-\frac{\sum_{i=1}^{N}{\left(y_{i}-{\hat{y}}_{i}\right)}^{2}}{\sum_{i=1}^{N}{\left(y_{i}-\bar{y}\right)}^{2}},\bar{y}=\frac{1}{N}\sum_{i=1}^{N}y_{i}$$

4. 95% Confidence Interval (CI): used to quantify the uncertainty of the mean of each metric, calculated as Equations 10–11.

(10) $${\text{CI}}_{95\%}=\bar{x}\pm t_{0.975,N-1}\cdot \frac{s}{\sqrt{N}}$$

(11) $$t=\frac{\bar{d}}{S_{d}/\sqrt{N}},d_{i}={\hat{y}}_{i}^{\mathrm{mod}el}-{\hat{y}}_{i}^{baseline},\bar{d}=\frac{1}{N}\sum_{i=1}^{N}d$$

Where N denotes the number of test samples, $y_{i}$ is the ground-truth count of the i-th sample, ${\hat{y}}_{i}$is the predicted count of the i-th sample, and $\bar{y}$is the mean of the ground-truth counts. s represents the sample standard deviation, $s_{d}$is the standard error of the mean, and $t_{0.975,N-1}$ is the critical value of the t-distribution with N−1 degrees of freedom.

Complexity is assessed through model parameters, floating point operations per second (FLOPs), and frames per second (FPS), indicating computational requirements. These combined metrics thus provide comprehensive insights into the model’s efficiency and accuracy for soybean stem detection. The corresponding formulas are given in Equations 12, 13:

(12) parameters=[r×(f×f)×o]+o

(13) $$FLOPs=2\times H_{out}\times W_{out}\times (C_{in}\times K^{2}\times bias)\times C_{out}$$

where r is the input size, f is the size of the convolution kernel, o is the output size, H×W is the size of the output feature map, $C_{in}$is the input channel, K is the kernel size, sis the stride, and $C_{out}$is the output channel.

These metrics reflect the model’s performance in the soybean seed counting task from multiple perspectives, providing a comprehensive evaluation of counting accuracy, robustness, goodness of fit, efficiency, and statistical significance.

## 3 Results and analysis

Experiments were conducted on a Linux workstation equipped with an NVIDIA RTX 4090 GPU (24 GB memory) and a 16-core Intel^®^ Xeon^®^ Gold 6430 processor, with 120 GB of system RAM. The deep learning framework was PyTorch, and Python 3.12 was used for programming. Input images were resized to 640 × 640 pixels with a batch size of 4. The model was trained using the Adam optimizer with an initial learning rate of 1×10^-4^, and a learning rate of 1×10^-5^ for the FPN module, over 200 epochs. A warm-up followed by a cosine decay learning rate schedule was employed, and gradient clipping with a maximum norm of 0.1 was applied to prevent gradient explosion. Model parameters were categorized into weight decay, no weight decay, and bias groups, with regularization implemented using L2 weight decay (1×10^-4^) and Dropout. Data augmentation during training included HSV color jittering, random horizontal flipping, Mosaic augmentation, and random affine transformations. During validation and testing, augmentation was disabled to ensure comparability and reproducibility. Training speed averaged ~4.5 iterations per second, processing approximately 18 images per second. Evaluation was performed every two epochs, with early stopping applied if no improvement was observed on the validation set.

### 3.1 Comparison analysis of experimental results

On the self-constructed single-plant soybean dataset, SoyCountNet was evaluated against six mainstream point-based counting methods, including P2PNet, DM-Count (Wang et al., 2020a), CSRNet (Li et al., 2018), BL (Ma et al., 2019), CAN (Liu et al., 2019), and FIDTM (Liang et al., 2022). These methods encompass the major point-counting strategies, such as direct point regression, density distribution matching, density-map regression, holistic CNN-based counting, and convolution–Transformer hybrid architectures. Model performance was evaluated using MAE, RMSE, and R², with 95% confidence intervals (CI) reported to quantify uncertainty (**Table 3**). The confidence intervals were computed based on paired statistical comparisons between SoyCountNet and each baseline method across the test samples, and statistical significance was assessed using a paired t-test at the 0.05 significance level.

**Table 3:** Comparison of the precision among different counting models.

Model	Backbone	MAE	RMSE	R²
SoyCountNet	*VGG19_BN*	4.61 ± 0.79 (3.63, 5.59)	6.03 ± 0.93 (4.87, 7.19)	0.94 ± 0.016 (0.92, 0.96)
*P2PNet*	*VGG19_BN*	9.20 ± 0.94 (8.03, 10.37)	11.80 ± 1.70 (9.69, 13.91)	0.78 ± 0.07 (0.69, 0.86)
*DM-Count*	*VGG19*	6.67 ± 0.68 (6.00, 7.34)	8.90 ± 1.22 (7.88, 9.92)	0.86 ± 0.05 (0.85, 0.95)
*CSRNet*	*VGG16*	16.33 ± 1.77 (14.71, 17.95)	20.15 ± 2.78 (17.37, 22.93)	0.64 ± 0.09 (0.55, 0.73)
*BL*	*VGG19*	10.35 ± 1.10 (9.30, 11.40)	12.56 ± 1.70 (11.10, 14.00)	0.73 ± 0.07 (0.66, 0.79)
*CAN*	*VGG16*	17.67 ± 1.81 (15.89, 19.44)	18.66 ± 2.57 (16.53, 20.78)	0.74 ± 0.07 (0.67, 0.80)
*FIDTM*	*VGG19*	9.80 ± 1.06 (8.83, 10.70)	14.51 ± 2.00 (12.51, 16.53)	0.78 ± 0.11 (0.67, 0.89)

Experimental results demonstrate that SoyCountNet consistently outperforms all baseline methods across all metrics. It achieves an MAE of 4.61 [95% CI 3.63, 5.59], approximately 30.9% lower than the best-performing baseline DM-Count (MAE 6.67 [95% CI 6.00, 7.34]), with an RMSE of 6.03 [95% CI 4.87, 7.19] and R² of 0.94 [95% CI 0.92, 0.96], indicating strong agreement between predicted and manually counted values. In contrast, traditional density-map regression models such as CSRNet and CAN perform poorly in dense or occluded regions, exhibiting substantially higher MAE and RMSE, which suggests their susceptibility to counting bias in complex field environments. P2PNet retains the advantage of point localization but yields lower counting accuracy (MAE 9.20, RMSE 11.80). DM-Count achieves lower MAE and RMSE than conventional CNN-based approaches but still accumulates errors in overlapping regions. FIDTM attains an R² comparable to P2PNet but exhibits higher MAE and RMSE, indicating limited robustness under challenging field conditions.

The superior performance of SoyCountNet can be attributed to its structural innovations: multi-scale feature extraction via VGG19_BN enhances feature representation; the SViT module enables global context modeling; the ECA mechanism strengthens channel attention; and the optimized loss function enforces spatial consistency. These components work synergistically to achieve precise counting and stable localization under dense, occluded, and complex backgrounds. Although the inference time slightly increases, the substantial improvements in counting accuracy and localization robustness highlight the strong potential of SoyCountNet for high-throughput field phenotyping and yield estimation applications.

### 3.2 Ablation experiments

To evaluate the effectiveness of each key component in SoyCountNet, a series of ablation experiments were conducted on the VGG19_BN backbone, loss function, SViT, and ECA modules. The modified P2PNet (denoted as P2PNet_VGG19_Loss, abbreviated as PV19_L) was used as the baseline model. Under identical datasets and training configurations, the SViT and ECA modules were independently incorporated for comparison, and the complete SoyCountNet was subsequently constructed. The results are presented in **Table 4**, where the best-performing configuration is highlighted in bold.

**Table 4:** Results of ablation experiments. Add various combinations of modules to the baseline model.

Model	MAE	MSE	RMSE	R²	Params(M)	FLOPs(G)	FPS
*PV16*	72.54 ± 14.86 (54.08, 91.00)	8010.44 ± 2897.67 (4410.70, 11611.18)	88.72 ± 14.46 (70.76, 106.68)	-11.46 ± 4.07 (-16.51, -6.41)	16.03	317.11	128.24
*PV19*	56.33 ± 11.10 (42.53, 70.13)	5070.12 ± 1564.87 (3125.70, 7014.54)	70.80 ± 11.23 (56.85, 84.75)	-6.91 ± 2.57 (-10.10, -3.72)	21.34	385.06	110.93
*PV16_L*	9.83 ± 1.03 (8.55, 11.11)	163.99 ± 47.36 (105.27, 222.71)	12.91 ± 1.68 (10.83, 14.99)	0.75 ± 0.064 (0.67, 0.82)	16.03	317.11	127.93
*PV19_L*	9.20 ± 0.94 (8.03, 10.37)	146.67 ± 44.06 (91.94, 201.40)	11.80 ± 1.70 (9.69, 13.91)	0.78 ± 0.07 (0.69, 0.86)	21.34	385.06	110.37
*PV19_L* *+SViT*	7.59 ± 0.84 (6.55, 8.63)	102.85 ± 31.53 (63.69, 142.01)	10.44 ± 1.47 (8.61, 12.27)	0.85 ± 0.06 (0.78, 0.93)	33.95	425.41	77.23
*PV19_L* *+ECA*	8.49 ± 0.48 (7.89, 9.09)	123.64 ± 18.46 (100.70, 146.58)	11.09 ± 0.79 (10.11, 12.07)	0.79 ± 0.05 (0.74, 0.85)	21.34	385.06	110.42
*PV19_L* *+SViT+ECA*	4.61 ± 0.79 (3.63, 5.59)	37.87 ± 12.14 (22.79, 52.95)	6.03 ± 0.93 (4.87, 7.19)	0.94 ± 0.016 (0.92, 0.96)	33.95	425.41	77.54

PV16 represents P2PNet_VGG16; PV19 represents P2PNet_VGG19; PV16_L and PV19_L denote P2PNet_VGG16 and P2PNet_VGG19 integrated with the proposed loss function, respectively.

As shown in **Table 4**, the baseline model PV19_L achieved an MAE of 9.20, RMSE of 11.80, and R² of 0.78, demonstrating basic counting capability but exhibiting significant errors in regions with high density or occlusion. Incorporating the SViT module reduced the MAE to 7.59, RMSE to 10.44, and increased R^2^ to 0.85, indicating that SViT effectively captures global context and long-range dependencies, thereby enhancing feature representation and semantic consistency. In contrast, the PV19_L+ECA model achieved an MAE of 8.49, RMSE of 11.09, and R^2^ of 0.79, showing only modest gains over the baseline. This suggests that the lightweight channel attention mechanism ECA contributes to emphasizing key features and suppressing background noise, but its effect is limited without the support of global modeling. Notably, several backbone variants, such as PV16 and PV16_L, perform noticeably worse, highlighting that backbone selection significantly impacts overall performance.

The fully integrated SoyCountNet, combining VGG19_BN, SViT, and ECA, achieved the best performance, with reductions of approximately 49.9% and 48.9% in MAE and RMSE, and a 20.5% improvement in R^2^ relative to the baseline. These results demonstrate the complementary effects of the modules: SViT reinforces global feature extraction, ECA enhances local channel responses, and the loss function constrains counting objectives. Together, these components synergistically improve accuracy, robustness, and stability across varying densities. The ablation study thus highlights the nuanced contributions of each module: some components (e.g., SViT) provide substantial improvement, others (e.g., ECA alone) offer moderate gains, and certain backbone choices can degrade performance, providing insight into the design rationale of SoyCountNet in complex field environments.

To further evaluate the lightweight design and deployability of SoyCountNet on intelligent agricultural platforms, different model configurations were assessed in terms of parameter, FLOPs, and FPS. The results indicate that PV16 exhibits lower Params and FLOPs and higher inference speed compared to PV19. Incorporating the ECA module into PV19_L does not significantly increase Params or FLOPs, nor does it noticeably reduce FPS, as the one-dimensional convolutional channel attention mechanism introduces negligible additional computation. Adding the SViT module increases Params and FLOPs to 33.95 M and 425.41 G, respectively, and reduces FPS to 77.54. Nevertheless, the model maintains single-frame inference within the sub-second range, satisfying real-time or near-real-time requirements for field phenotyping and intelligent agricultural applications. Overall, these results demonstrate that SoyCountNet remains lightweight and suitable for practical deployment.

### 3.3 Visualization results and analysis of soybean pod counting

**Figure 8:**
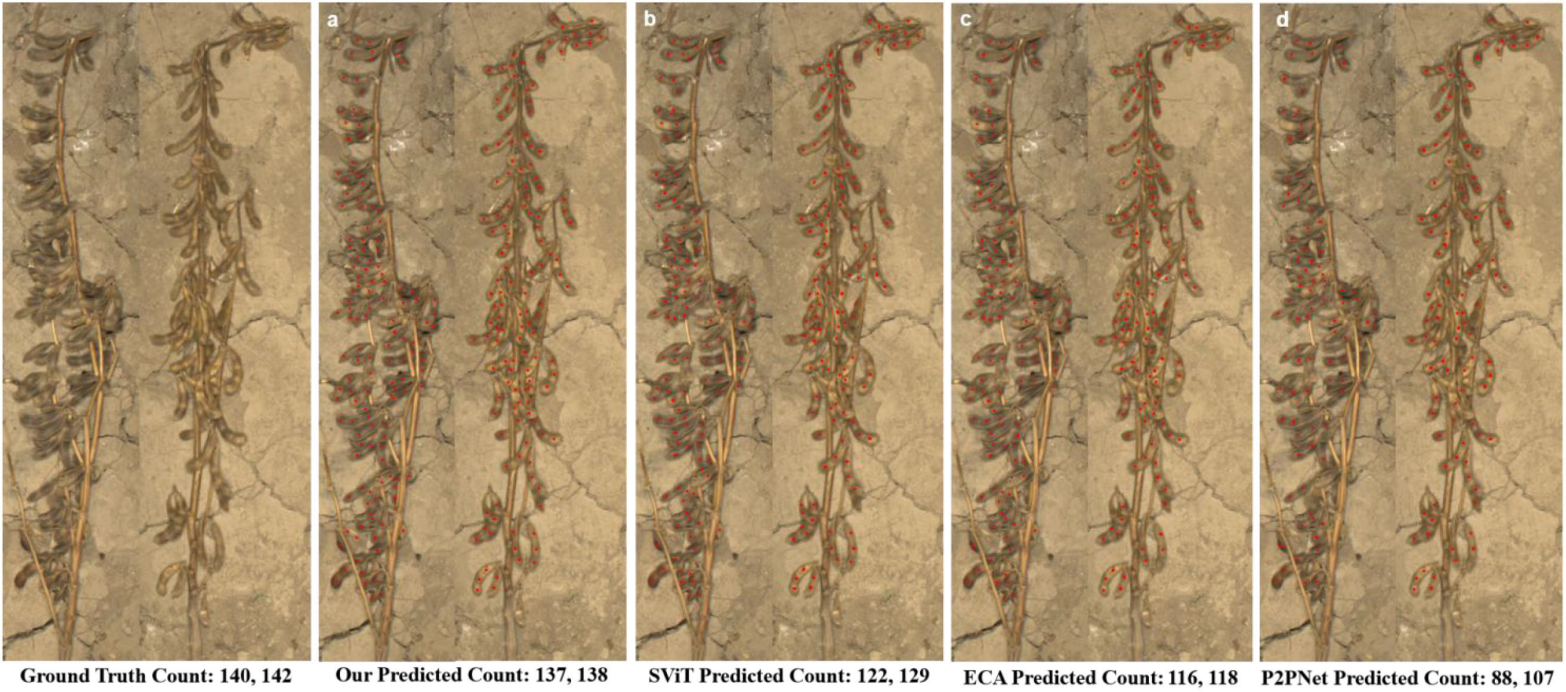
Visualization of prediction results from different counting models: **(a)** SoyCountNet, **(b)** PV19_L+SViT, **(c)** PV19_L+ECA, and **(d)** PV19_L.

As shown in **Figure 8**, this study visualized and compared the prediction results of the baseline model (PV19_L) and its improved versions (PV19_L+SViT and PV19_L+ECA) on selected single-plant soybean test samples. The results indicate that the traditional point-based P2PNet exhibits significant limitations in single-plant soybean seed counting, prone to both missed and false detections. On plants with minimal occlusion, P2PNet can achieve relatively accurate counts; however, in densely seeded or heavily occluded regions, the counting errors and standard deviations increase substantially, reflecting insufficient robustness to complex backgrounds.

The PV19_L+SViT model, incorporating the SViT module, captures both local and global features during extraction, effectively improving recognition of dense and occluded seeds. Visualization results show that this model significantly reduces repeated missed detections among adjacent seeds and achieves higher detection accuracy in mildly occluded areas, demonstrating enhanced anti-interference capability and generalization. Moreover, the PV19_L+ECA model, integrating the ECA channel attention mechanism, adaptively adjusts feature channel weights to highlight information relevant to seed counting while suppressing background noise. Experimental observations indicate that this model maintains stable counting performance under severe occlusion and complex backgrounds, further reducing missed detection rates and producing predictions closer to the true distribution.

**Figure 9:**
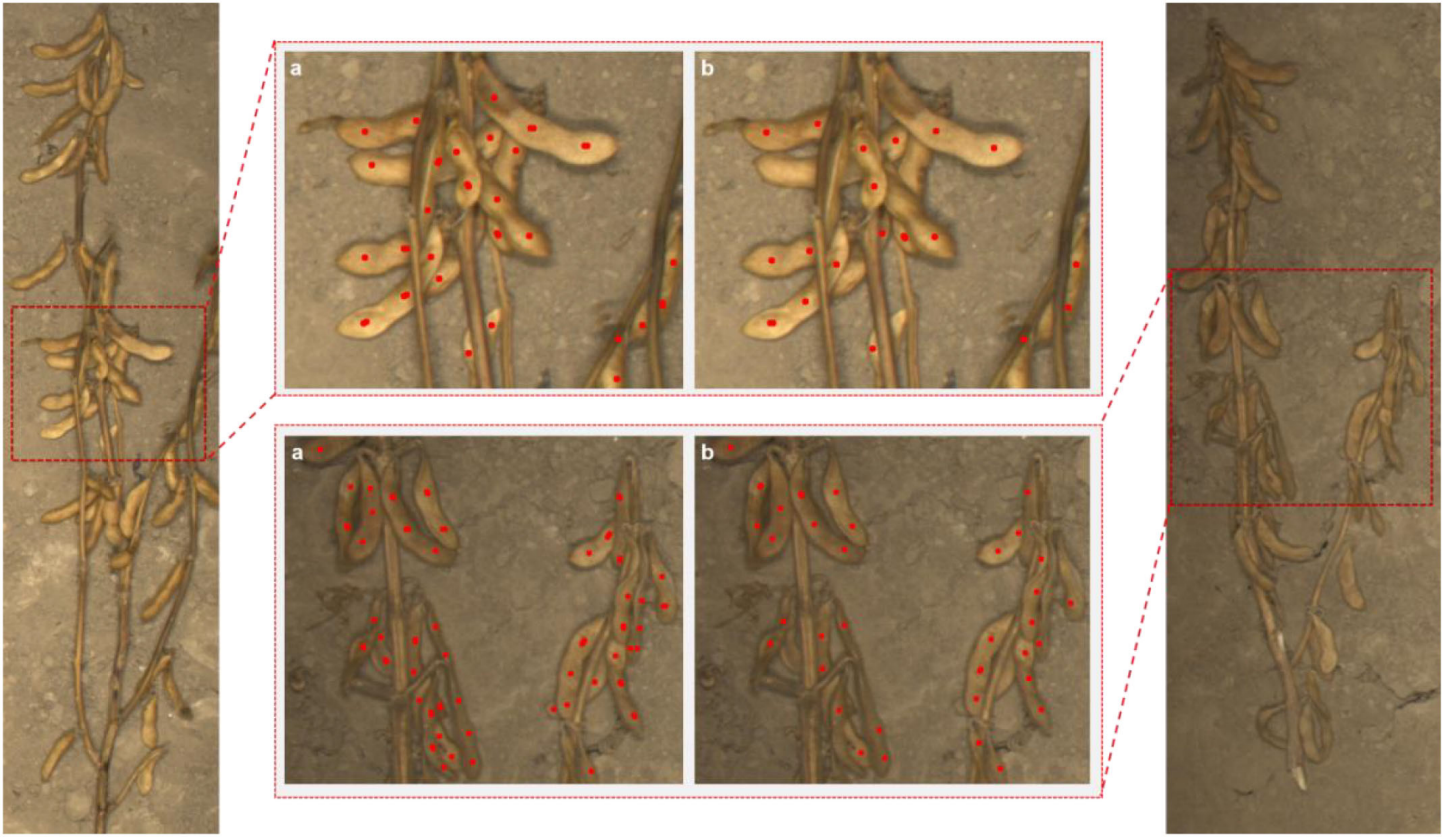
Comparison of counting results between **(a)** SoyCountNet and **(b)** PV19_L.

**Figure 9** provides a more detailed view of model performance in fine-grained regions. Compared with PV19_L, SoyCountNet substantially reduces missed detections in local areas. In the magnified regions, PV19_L still misses some seeds under partial occlusion or uneven seed distribution, whereas SoyCountNet accurately identifies these seeds. Additionally, SoyCountNet maintains stable performance under varying occlusion levels and uneven seed development, demonstrating strong noise resistance and feature robustness. Overall, both the global visualization and local detail analysis further validate the effectiveness and stability of SoyCountNet for high-precision single-plant soybean seed counting in complex field environments.

**Figure 10:**
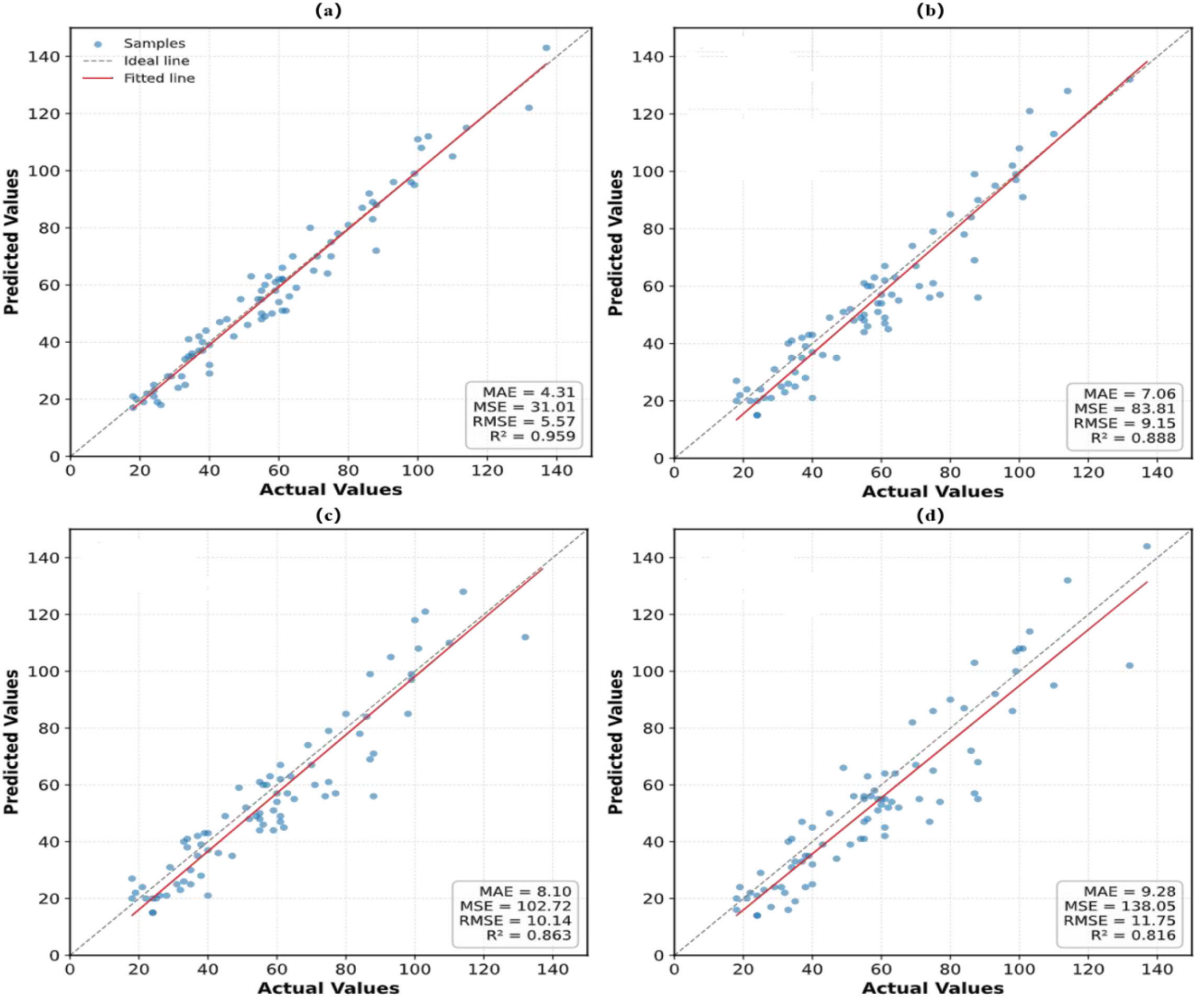
Fitting analysis of predicted soybean grain numbers and ground truth values for different models: **(a)** SoyCountNet, **(b)** P2PNetVGG19_BN+SViT, **(c)** P2PNetVGG19_BN+ECA, **(d)** PV19_L.

Furthermore, the predicted results of different models were fitted against the ground truth on the test set, as shown in **Figure 10**. The fitting curves clearly illustrate differences in counting performance among the models. SoyCountNet exhibits the highest agreement between predicted and true values, achieving the best overall performance, indicating strong robustness and reliability in complex field conditions and meeting practical requirements for single-plant seed counting. In contrast, the baseline PV19_L demonstrates weaker fitting performance and larger prediction deviations, highlighting the limitations of traditional point-based counting methods under complex backgrounds. By incorporating the SViT and ECA modules, SoyCountNet enhances feature extraction and channel attention, significantly improving adaptability and counting accuracy.

### 3.4 Generalization performance across different soybean varieties

Soybean varieties exhibit significant differences in pod morphology, color, pod position, and density. Therefore, the performance of a single-plant seed counting model under cross-varietal conditions is an important indicator of its generalization capability. To evaluate the adaptability of SoyCountNet across diverse genetic backgrounds, six representative salt-tolerant soybean varieties were selected as test samples. For each variety, twenty images were randomly chosen for validation, and the prediction results are summarized in **Table 5**. The results indicate that SoyCountNet maintains high counting accuracy across all varieties, with R² values ranging from 0.87 to 0.95, demonstrating stability and robustness under cross-varietal conditions. Among these, D3 and D4 exhibited the best performance, indicating that the model can accurately capture the phenotypic characteristics of these varieties and achieve consistent seed counting. D1 and D2 also showed high consistency with relatively small prediction errors, confirming the model’s reliability for varieties with moderate morphological variation. In contrast, D5 and D6 had slightly lower R² values and higher RMSE, reflecting certain prediction deviations, which may be attributed to pod overlap, color similarity with the background, or edge blurring caused by light reflection.

**Table 5:** Counting accuracy of SoyCountNet on different soybean varieties.

Number	D1	D2	D3	D4	D5	D6
Variety	Qihuang34	Qingnong Bean 2312	AFA1	AFA1	AFA2	Qingnong Bean 2306
MAE	3.60	3.20	4.10	3.90	4.65	4.40
SMSE	4.48	3.92	4.64	4.77	5.73	5.39
R^2^	0.90	0.92	0.95	0.95	0.90	0.87

The visualization results in **Figure 9** further support these findings. Overall, the predicted values exhibit a strong linear correlation with ground-truth values, and most scatter points are closely aligned along the diagonal, indicating high consistency between model predictions and manual annotations. D3 and D4 displayed the most compact scatter distributions with minimal deviation, demonstrating superior stability and agreement, while D1 and D2 were similarly well-aligned, indicating accurate counting for samples with regular pod morphology and clear background contrast. By comparison, some points for D5 and D6 fall slightly below the diagonal, suggesting minor underestimation, likely due to pod clustering or complex illumination conditions reducing local feature responses.

**Figure 11:**
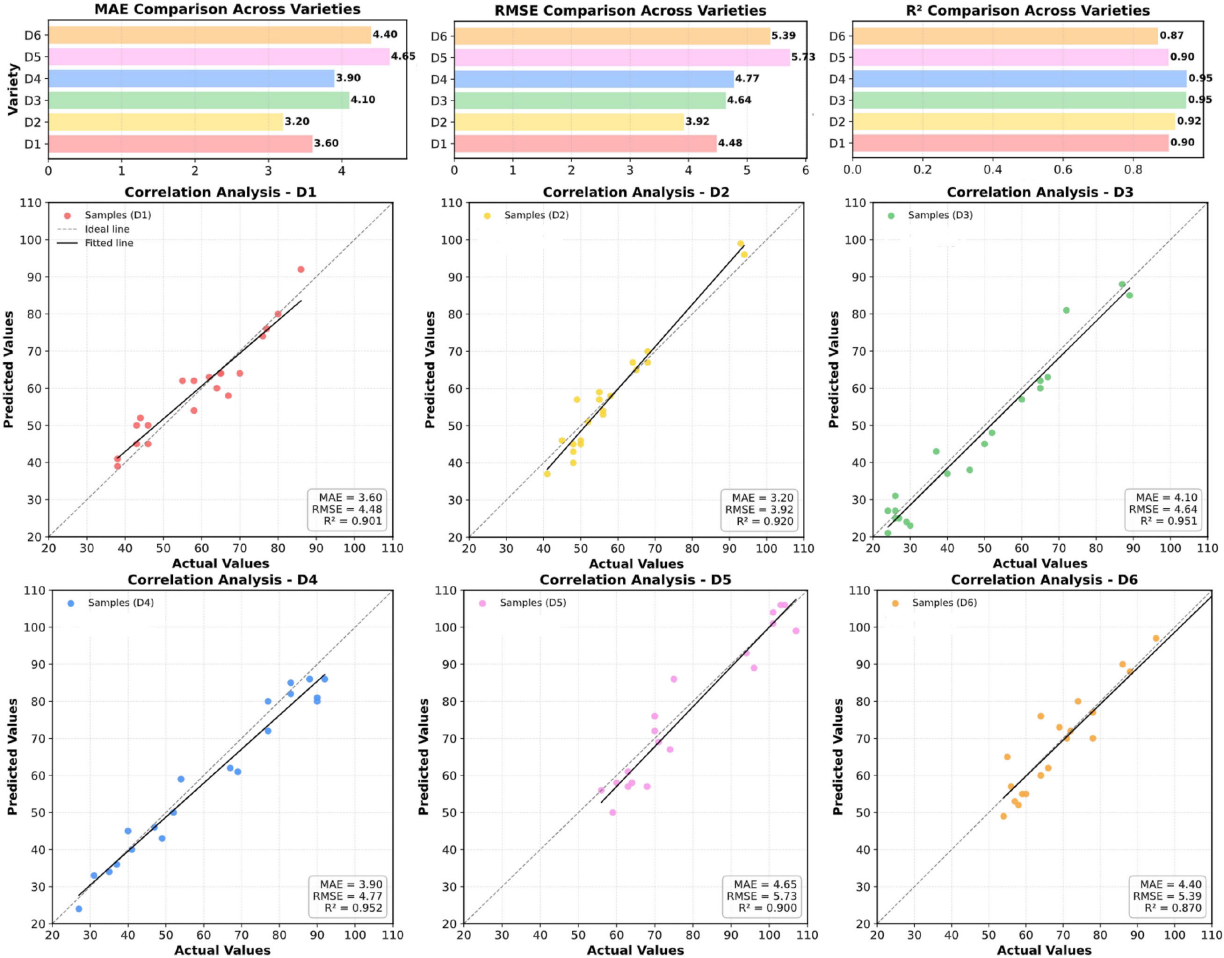
SoyCountNet detection performance across different soybean varieties.

As shown in **Figure 11**, Bar chart comparisons show that although MAE and RMSE vary slightly among different varieties, the overall range is limited (MAE variation within 1.5), indicating consistent performance in cross-varietal transfer. This suggests that the deep semantic features learned by SoyCountNet are highly transferable and not restricted to specific morphological patterns. The feature extraction module (VGG19_SN + SViT) and channel attention mechanism (ECA) effectively focus on seed regions while suppressing background interference, enabling reliable performance under diverse phenotypic conditions. In summary, SoyCountNet demonstrates robust feature representation and generalization ability across multiple soybean varieties, maintaining high-precision seed counting under varying morphological and environmental conditions. This indicates its potential for large-scale, multi-variety field phenotyping and provides reliable methodological support for intelligent yield estimation and varietal evaluation.

**Figure 12:**
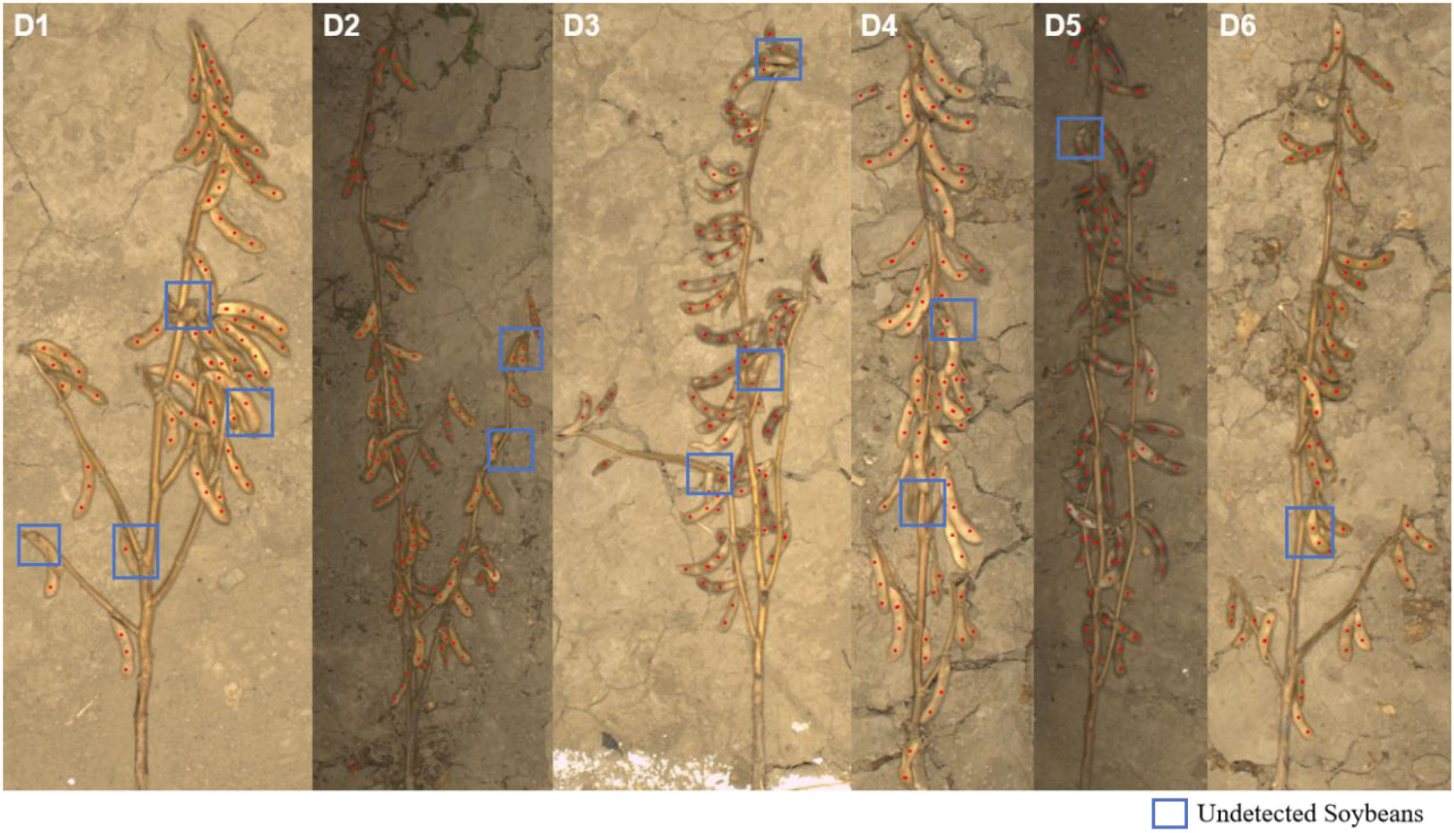
SoyCountNet detection results and missed detections across different soybean varieties (blue boxes indicate missed detections).

As shown in **Figure 12**, SoyCountNet still exhibits certain counting errors under complex field conditions, primarily in the form of missed detections (highlighted by blue boxes). These missed detections generally occur in regions with dense pod overlap or low contrast between pods and the background, where foreground features are difficult to distinguish. Additionally, uneven illumination, shadow occlusion, and surface reflections may lead to information loss during feature extraction, affecting counting completeness. Future work could focus on integrating adaptive feature enhancement modules, cross-scale feature alignment mechanisms, or illumination-invariant enhancement strategies to further improve the model’s robustness and generalization in complex field environments.

**Figure 13:**
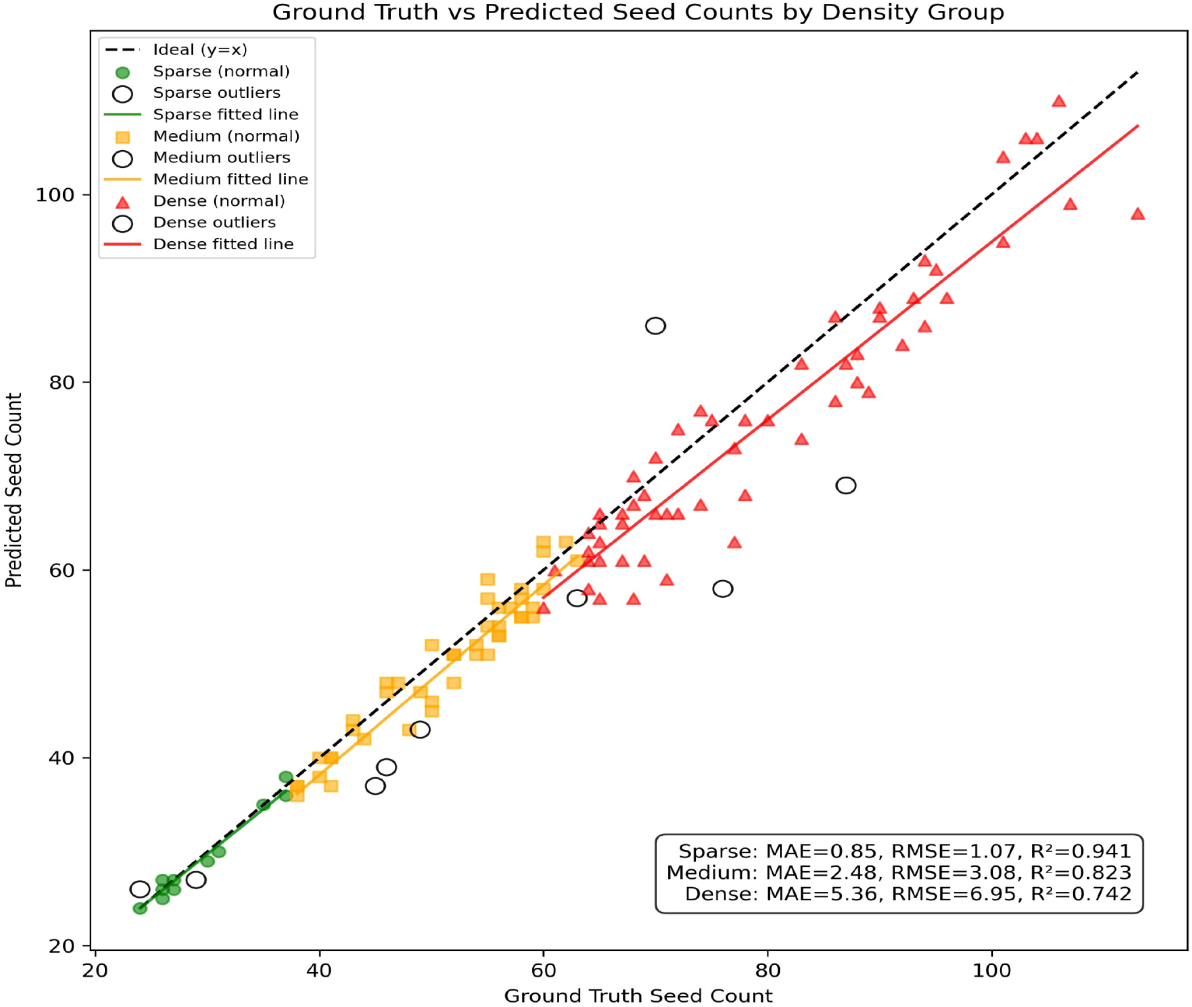
Scatter plots of ground-truth vs. predicted seed counts for different density groups (Sparse, Medium, and Dense).

Additionally, the dataset of 120 samples from six cultivars was stratified into three groups based on seed density: Sparse, Medium, and Dense. **Figure 13** presents scatter plots of the ground-truth versus predicted values for each group, with overlaid fitted lines and the global ideal reference line (y = x). Quantitative analysis reveals that the model performs best on sparse samples (MAE = 0.85, RMSE = 1.07, R² = 0.941), shows moderate accuracy on medium-density samples (MAE = 2.48, RMSE = 3.08, R² = 0.823), and exhibits larger deviations on dense samples (MAE = 5.36, RMSE = 6.95, R² = 0.742). The fitted lines indicate a slight underestimation as seed density increases, but overall, the model remains well-aligned with the ideal line, demonstrating its robustness across different density levels. The results suggest that SoyCountNet achieves high-precision counting under sparse to medium-density conditions, while its performance deteriorates under high-density conditions with heavy occlusion, highlighting areas for future improvement.

## 4 Discussion

### 4.1 Architectural advantages and performance gains

Through the synergistic integration of the VGG19_BN, SViT, and ECA modules with a spatial distribution–constrained loss function, SoyCountNet achieves notable improvements in soybean seed counting accuracy under complex field conditions. Quantitative evaluations show that SoyCountNet reduces MAE by approximately 49.9% and 30.9% compared with P2PNet and DM-Count, respectively. The non-overlapping 95% confidence intervals of MAE between SoyCountNet and the best baseline indicate a statistically significant improvement (p< 0.05). At the architectural level, the three core modules play complementary roles: VGG19_BN provides multi-scale, fine-grained feature representations as a high-resolution basis for semantic modeling; SViT captures long-range dependencies to alleviate feature confusion in dense and occluded regions; and ECA adaptively reweights channel responses to enhance target discrimination and suppress background interference. Additionally, the proposed Near-Point and Overlap Penalties explicitly constrain the spatial distribution of predicted points, reducing redundancy and aggregation, thereby improving both counting accuracy and spatial consistency. Unlike conventional object detection frameworks (Vijayakumar and Vairavasundaram, 2024; Muzammul and Li, 2025), SoyCountNet employs a point-level supervision paradigm that enables simultaneous counting and localization without bounding-box annotations (Zheng et al., 2023). This paradigm significantly reduces annotation cost and training complexity, while promoting finer spatial alignment between predicted density peaks and seed centroids. Its end-to-end architecture eliminates post-processing steps such as segmentation, filtering, and non-maximum suppression (NMS), thereby enhancing efficiency and facilitating real-time, high-throughput field phenotyping. In terms of generalization, SoyCountNet maintains stable performance across six soybean cultivars, with MAE variation within 1.5, demonstrating robust cross-varietal adaptability and environmental resilience. Overall, SoyCountNet achieves a well-balanced improvement in counting accuracy, localization precision, and computational efficiency through coordinated architectural and loss design, providing a reliable and scalable framework for intelligent breeding and precision agriculture. Its architecture and training paradigm can further be extended to other crop phenotyping applications requiring dense object localization.

### 4.2 Occlusion problem and mitigation strategies

In field conditions, soybean pods, stems, and leaves are often densely intertwined, resulting in varying degrees of pod and stem occlusion. Such occlusion obscures seed texture and boundary features, thereby degrading the model’s counting accuracy and stability (Wu et al., 2025). To address these challenges, this study introduces a series of targeted optimization strategies from both architectural and data perspectives. First, inspired by the point-based density estimation paradigm of P2PNet, the soybean seed counting task is reformulated from an explicit detection problem into a density regression problem. By modeling each seed center as a Gaussian kernel, the network achieves a continuous representation of the spatial target distribution. This formulation enables the model to infer potential targets in occluded regions through neighborhood feature aggregation, thereby maintaining high counting accuracy and effectively reducing missed detections even under severe pod overlap or partial occlusion (Wang et al., 2025). Second, at the feature modeling level, the SViT module is introduced to strengthen long-range semantic dependencies across spatial dimensions. Through its self-attention mechanism, SViT adaptively redistributes spatial feature weights, allowing the network to more effectively perceive contextual cues in occluded regions and recover the semantic integrity of partially hidden pods. Meanwhile, the ECA module enhances feature discriminability in dense seed regions by emphasizing informative channels and suppressing redundant background noise. Finally, at the data level, an occlusion-aware data augmentation strategy is designed to generate training samples with varying occlusion ratios and spatial configurations, thereby improving the model’s generalization and robustness under real-world field conditions. In summary, the proposed multi-level occlusion mitigation framework—encompassing structural design, feature enhancement, and data augmentation—forms a synergistic optimization mechanism. This framework enables SoyCountNet to maintain high counting accuracy and stability even in densely occluded scenarios, providing a reliable technical foundation for high-throughput and automated soybean phenotyping.

### 4.3 Limitations and mitigation strategies

Maintaining consistent cultivation and management practices—such as planting density, fertilization, and irrigation—is critical for data comparability and reproducibility (Angidi et al., 2025). The relatively low metrics observed here largely reflect the stress imposed by saline-alkali soil on soybean physiology, which reduces root uptake and photosynthetic efficiency, thereby decreasing pod number and yield (Hasanuzzaman et al., 2022). Although SoyCountNet performed robustly under these conditions, its accuracy may still be affected by soil salinity fluctuations. For image acquisition, a ground-based platform enabled close-range photography with ease of deployment and operational efficiency, suitable for small-scale field experiments. However, imaging is constrained by plant height and canopy density, often requiring manual adjustment of camera angles or plant manipulation to expose pods, limiting data collection flexibility and continuity (Xu et al., 2023). The current platform offers only three degrees of freedom and relies on manual assistance. Future improvements may involve multi-degree-of-freedom systems, such as adjustable 3D gimbals or robotic arms, to enable multi-angle, non-destructive imaging and enhance model transferability. For dataset construction, point-level annotations were used instead of polygonal or bounding-box labels, reducing labeling time and cost by roughly 80% while providing supervision suited for dense counting tasks (Xu et al., 2025). Nonetheless, minor human errors, such as missed or slightly shifted points, may subtly affect density learning and spatial modeling. Semi-automatic annotation combined with active learning could iteratively refine labels, improving consistency and accuracy. Overall, SoyCountNet achieves high-precision, robust soybean seed counting under *in situ* conditions, though data acquisition flexibility, environmental adaptability, and annotation consistency remain areas for improvement. Future work will explore multimodal data fusion, high-throughput dynamic phenotyping, and weakly supervised learning to advance intelligent, automated, and generalizable crop phenotyping systems.

### 4.4 Implications for future applications and extensions

Building on the identified limitations, SoyCountNet provides a novel technical framework and practical approach for addressing the challenges of automatic crop counting in complex field environments. Its lightweight architecture and strong generalization capability enable real-time deployment across diverse intelligent agricultural platforms, including mobile imaging systems, ground robots, and automated phenotyping stations, thereby significantly enhancing the efficiency and timeliness of field data acquisition and phenotypic analysis. Future improvements can be pursued along three main directions: data acquisition, feature modeling, and cross-domain transfer. First, in data acquisition, multi-degree-of-freedom imaging systems can enable multi-angle, non-destructive *in situ* capture, mitigating occlusion and incomplete visual information while enhancing model adaptability and robustness under complex natural conditions. Second, in challenging environments such as saline-alkali fields, where data distributions are uneven and visual features are degraded, integrating multimodal data sources (e.g., multispectral, infrared, or depth images) is recommended to improve environmental robustness and yield estimation reliability (Singh et al., 2024). In terms of annotation and model training, semi-supervised, self-supervised, or active learning strategies can reduce reliance on manual labeling through iterative human–model interaction. These approaches can mitigate annotation bias, improve generalization, and facilitate the construction of larger and more diverse datasets (Nivetha et al., 2025). Furthermore, incorporating additional soybean cultivars in future studies could provide a more comprehensive evaluation of SoyCountNet’s generalization ability. In addition, cross-crop transfer learning—from soybean to other crops such as rice, maize, and wheat—could also be explored for applicability beyond soybean. Finally, systematic research into real-time optimization and multimodal fusion mechanisms may lay the foundation for a unified intelligent phenotyping framework. Through large-scale validation across diverse ecological conditions, SoyCountNet has the potential to evolve into a core technological tool linking field observation with intelligent decision-making, thereby advancing precision agriculture, digital breeding, and sustainable crop production (Visakh et al., 2024).

## 5 Conclusion

Accurate counting of soybean seeds per plant is critical for yield estimation and cultivar evaluation. This study presents SoyCountNet, a deep learning framework for non-destructive, *in situ* counting and localization of individual soybean seeds under field conditions, built upon a self-developed high-throughput phenotyping platform. The model integrates VGG19_BN backbone, SViT, and ECA modules, enabling effective capture of both local and global features while mitigating occlusion from stems and pods and feature loss due to incomplete seed development. An improved loss function incorporating nearest-neighbor and target-overlap penalties further enhances counting accuracy and spatial localization consistency. Experimental results demonstrate that SoyCountNet significantly outperforms existing methods on the in-house field soybean dataset, achieving a mean absolute error of 4.61 (95% CI: 3.63–5.59) and a coefficient of determination of 0.94 (95% CI: 0.92–0.96), with robust performance across different cultivars, providing reliable support for yield prediction and precision breeding. The lightweight architecture and strong generalization ability of SoyCountNet enable deployment across diverse intelligent agricultural platforms. Future integration with multi-view imaging, multimodal sensing, and cross-crop transfer learning could further improve robustness, adaptability, and real-time performance, supporting the development of unified intelligent phenotyping systems, automated yield estimation, and sustainable precision agriculture.

## Data Availability

The data analyzed in this study is subject to the following licenses/restrictions: The dataset generated and analyzed during the current study is not publicly available due to institutional policy and ongoing related research, but it can be made available from the corresponding author upon reasonable request. Requests to access these datasets should be directed to FL, dbkh@qau.edu.cn.

## References

[B1] Angidi S. Madankar K. Tehseen M. M. Bhatla A. (2025). Advanced high-throughput phenotyping techniques for managing abiotic stress in agricultural crops—A comprehensive review. Crops 5, 8. doi: 10.3390/crops5020008

[B2] Behera T. K. Bakshi S. Nappi M. Sa P. K. (2023). Superpixel-based multiscale CNN approach toward multiclass object segmentation from UAV-captured aerial images. IEEE Journal of Selected Topics in Applied Earth Observations and Remote Sensing. 16, 1771–1784. doi: 10.1109/JSTARS.2023.3239119

[B3] Chen Z. Wang J. Jin J. (2023). Fully automated proximal hyperspectral imaging system for high-resolution and high-quality *in vivo* soybean phenotyping. Precis. Agric. 24, 2395–2415. doi: 10.1007/s11119-023-10045-5

[B4] Fan J. Zhang Y. Wen W. Gu S. Lu X. Guo X. (2021). The future of Internet of Things in agriculture: Plant high-throughput phenotypic platform. J. Cleaner Production 280, 123651. doi: 10.1016/j.jclepro.2020.123651

[B5] Han K. Wang Y. Chen H. Chen X. Guo J. Liu Z. . (2022). A survey on vision transformer. IEEE Trans. Pattern Anal. Mach. Intell. 45, 87–110. doi: 10.1109/TPAMI.2022.3152247, PMID: 35180075

[B6] Hasanuzzaman M. Parvin K. Anee T. I. Masud A. A. C. Nowroz F. (2022). “ Salt stress responses and tolerance in soybean,” in Plant Stress Physiology – Perspectives in Agriculture. (London, UK: IntechOpen).

[B7] Jin C. Zhou L. Pu Y. Zhang C. Qi H. Zhao Y. (2025). Application of deep learning for high-throughput phenotyping of seed: a review. Artif. Intell. Rev. 58, 76. doi: 10.1007/s10462-024-11079-5

[B8] Kwon H. Lee S. H. Kim M. Y. Ha J. (2025). Development of an automated phenotyping platform and identification of a novel QTL for drought tolerance in soybean. Plant Phenomics. 100102. doi: 10.1016/j.plaphe.2025.100102, PMID: 41416200 PMC12709953

[B9] Li M. Liu Y. Wang C. Yang X. Li D. Zhang X. . (2020). Identification of traits contributing to high and stable yields in different soybean varieties across three Chinese latitudes. Front. Plant Sci. 10, 1642. doi: 10.3389/fpls.2019.01642, PMID: 32038668 PMC6985368

[B10] Li J. Magar R. T. Chen D. Lin F. Wang D. Yin X. . (2024). SoybeanNet: Transformer-based convolutional neural network for soybean pod counting from Unmanned Aerial Vehicle (UAV) images. Comput. Electron. Agric. 220, 108861. doi: 10.1016/j.compag.2024.108861

[B11] Li Y. Zhang X. Chen D. (2018). “ Csrnet: Dilated convolutional neural networks for understanding the highly congested scenes,” in Proceedings of the IEEE conference on computer vision and pattern recognition. 1091–1100.

[B12] Liang D. Xu W. Zhu Y. Zhou Y. (2022). Focal inverse distance transform maps for crowd localization. IEEE Trans. Multimedia 25, 6040–6052. doi: 10.1109/TMM.2022.3203870

[B13] Liu W. Salzmann M. Fua P. (2019). “ Context-aware crowd counting,” in Proceedings of the IEEE/CVF conference on computer vision and pattern recognition. 5099–5108.

[B14] Liu F. Wang S. Pang S. Han Z. Zhao L. (2025a). SmartPod: an automated framework for high-precision soybean pod counting in field phenotyping. Agronomy 15, 791. doi: 10.3390/agronomy15040791

[B15] Liu F. Wang S. Zhao L. (2025b). Research progress and prospect of intelligent high-throughput crop phenotyping platform. J. Crop Health 77, 156. doi: 10.1007/s10343-025-01228-3

[B16] Ma Z. Wei X. Hong X. Gong Y. (2019). “ Bayesian loss for crowd count estimation with point supervision,” in Proceedings of the IEEE/CVF international conference on computer vision. 6142–6151.

[B17] Mishra R. Tripathi M. K. Sikarwar R. S. Singh Y. Tripathi N. (2024). Soybean (Glycine max L. Merrill): A multipurpose legume shaping our world. Plant Cell Biotechnol. Mol. Biol. 25, 17–37. doi: 10.56557/pcbmb/2024/v25i3-48643

[B18] Moeinizade S. Pham H. Han Y. Dobbels A. Hu G. (2022). An applied deep learning approach for estimating soybean relative maturity from UAV imagery to aid plant breeding decisions. Mach. Learn. Appl. 7, 100233. doi: 10.1016/j.mlwa.2021.100233

[B19] Murphy K. M. Ludwig E. Gutierrez J. Gehan M. A. (2024). Deep learning in image-based plant phenotyping. Annu. Rev. Plant Biol. 75, 771–795. doi: 10.1146/annurev-arplant-070523-042828, PMID: 38382904

[B20] Muzammul M. Li X. (2025). Comprehensive review of deep learning-based tiny object detection: challenges, strategies, and future directions. Knowledge Inf. Syst. 67, 3825–3913. doi: 10.1007/s10115-025-02375-9

[B21] Nivetha R. Sriharipriya K. C. Balusamy B. (2025). Self-supervised learning graphical neural network driven prediction model for path-planning and navigation in smart sustainable agriculture. IEEE Access. 13, 151235–151257. doi: 10.1109/ACCESS.2025.3602476

[B22] Okada M. Barras C. Toda Y. Hamazaki K. Ohmori Y. Yamasaki Y. . (2024). High-throughput phenotyping of soybean biomass: conventional trait estimation and novel latent feature extraction using UAV remote sensing and deep learning models. Plant Phenomics. 6, 0244. doi: 10.34133/plantphenomics.0244, PMID: 39252878 PMC11382017

[B23] Singh R. Nisha R. Naik R. Upendar K. Nickhil C. Deka S. C. (2024). Sensor fusion techniques in deep learning for multimodal fruit and vegetable quality assessment: A comprehensive review. J. Food measurement characterization 18, 8088–8109. doi: 10.1007/s11694-024-02789-z

[B24] Tian C. Wang J. Zheng D. Li Y. Zhang X. (2025). Oat Ears Detection and Counting Model in Natural Environment Based on Improved Faster R-CNN. Agronomy. 15:3, 536.

[B25] Vijayakumar A. Vairavasundaram S. (2024). Yolo-based object detection models: A review and its applications. Multimedia Tools Appl. 83, 83535–83574. doi: 10.1007/s11042-024-18872-y

[B26] Visakh R. L. Anand S. Reddy S. B. Jha U. C. Sah R. P. Beena R. (2024). Precision phenotyping in crop science: from plant traits to gene discovery for climate-smart agriculture. Plant Breed. 0, 1–29 doi: 10.1111/pbr.13228

[B27] Wang B. Liu H. Samaras D. Nguyen M. H. (2020a). Distribution matching for crowd counting. Adv. Neural Inf. Process. Syst. 33, 1595–1607.

[B28] Wang Q. Wu B. Zhu P. Li P. Zuo W. Hu Q. (2020b). “ ECA-Net: Efficient channel attention for deep convolutional neural networks,” in Proceedings of the IEEE/CVF conference on computer vision and pattern recognition. 11534–11542.

[B29] Wang M. Zhou X. Chen Y. (2025). A comprehensive survey of crowd density estimation and counting. IET Image Process. 19, e13328. doi: 10.1049/ipr2.13328

[B30] Wattana M. Siriluk B. Khotwit S. (2018). Counting and separating damaged seeds of soybean seeds using image processing. Int. J. Advanced Science Eng. Inf. Technol. 8, 1366–1371. doi: 10.18517/ijaseit.8.4.6513

[B31] Wei B. Ma X. Guan H. Yu M. Yang C. He H. . (2023). Dynamic simulation of leaf area index for the soybean canopy based on 3D reconstruction. Ecol. Inf. 75, 102070. doi: 10.1016/j.ecoinf.2023.102070

[B32] Wu Q. Liu F. Han Z. Wang H. Liu H. Xin N. . (2025). SPCNet: an intelligent field-based soybean seed counting algorithm for salinity stress response evaluation. J. Crop Health 77, 145. doi: 10.1007/s10343-025-01215-8

[B33] Xu X. Geng Q. Gao F. Xiong D. Qiao H. Ma X. (2023). Segmentation and counting of wheat spike grains based on deep learning and textural feature. Plant Methods 19, 77. doi: 10.1186/s13007-023-01062-6, PMID: 37528413 PMC10394929

[B34] Xu B. Zhang J. Tang Z. Zhang Y. Xu L. Lu H. . (2025). Nighttime environment enables robust field-based high-throughput plant phenotyping: A system platform and a case study on rice. Comput. Electron. Agric. 235, 110337. doi: 10.1016/j.compag.2025.110337

[B35] Yang S. Zheng L. Wu T. Sun S. Zhang M. Li M. . (2024). High-throughput soybean pods high-quality segmentation and seed-per-pod estimation for soybean plant breeding. Eng. Appl. Artif. Intell. 129, 107580. doi: 10.1016/j.engappai.2023.107580

[B36] Yu C. Feng J. Zheng Z. Guo J. Hu Y. (2024). A lightweight SOD-YOLOv5n model-based winter jujube detection and counting method deployed on Android. Computers and Electronics in Agriculture. 218, 108701. doi: 10.1016/j.compag.2024.108701

[B37] Zavafer A. Bates H. Mancilla C. Ralph P. J. (2023). Phenomics: conceptualization and importance for plant physiology. Trends Plant Sci. 28, 1004–1013. doi: 10.1016/j.tplants.2023.03.023, PMID: 37137749

[B38] Zhang Q. Y. Fan K. J. Tian Z. Guo K. Su W. H. (2024). High-Precision automated soybean phenotypic feature extraction based on deep learning and computer vision. Plants 13, 2613. doi: 10.3390/plants13182613, PMID: 39339587 PMC11435354

[B39] Zhang Z. Jin X. Rao Y. Wan T. Wang X. Li J. . (2024). DSBEAN: An innovative framework for intelligent soybean breeding phenotype analysis based on various main stem structures and deep learning methods. Comput. Electron. Agric. 224, 109135. doi: 10.1016/j.compag.2024.109135

[B40] Zhang C. Zha B. Yuan R. Zhao K. Sun J. Liu X. . (2025). Identification of quantitative trait loci for node number, pod number, and seed number in soybean. Int. J. Mol. Sci. 26, 2300. doi: 10.3390/ijms26052300, PMID: 40076921 PMC11900990

[B41] Zhao J. Kaga A. Yamada T. Komatsu K. Hirata K. Kikuchi A. . (2023). Improved field-based soybean seed counting and localization with feature level considered. Plant Phenomics 5, 0026. doi: 10.34133/plantphenomics.0026, PMID: 36939414 PMC10019992

[B42] Zheng H. Fan X. Bo W. Yang X. Tjahjadi T. Jin S. (2023). A multiscale point-supervised network for counting maize tassels in the wild. Plant Phenomics 5, 0100. doi: 10.34133/plantphenomics.0100, PMID: 37791249 PMC10545326

[B43] Zhou L. Han D. Sun G. Liu Y. Yan X. Jia H. . (2025). Soybean yield estimation and lodging discrimination based on lightweight UAV and point cloud deep learning. Plant Phenomics. 7, 100028. doi: 10.1016/j.plaphe.2025.100028, PMID: 41415158 PMC12710009

